# Biocide Susceptibility and Antimicrobial Resistance of *Escherichia coli* Isolated from Swine Feces, Pork Meat and Humans in Germany

**DOI:** 10.3390/antibiotics12050823

**Published:** 2023-04-27

**Authors:** David Attuy Vey da Silva, Ralf Dieckmann, Oliwia Makarewicz, Anita Hartung, Astrid Bethe, Mirjam Grobbel, Vitaly Belik, Mathias W. Pletz, Sascha Al Dahouk, Szilvia Neuhaus

**Affiliations:** 1 Department of Biological Safety, German Federal Institute for Risk Assessment, 10589 Berlin, Germany; david-attuy-vey.da-silva@bfr.bund.de (D.A.V.d.S.);; 2 Department of Veterinary Medicine, Freie Universität Berlin, 14163 Berlin, Germany; 3 Institute for Infectious Diseases and Infection Control, Jena University Hospital, 07747 Jena, Germany; 4 Institute of Microbiology and Epizootics, Centre for Infection Medicine, Department of Veterinary Medicine, Freie Universität Berlin, 14163 Berlin, Germany; 5 Veterinary Centre for Resistance Research (TZR), Freie Universität Berlin, 14163 Berlin, Germany; 6 System Modeling Group, Institute of Veterinary Epidemiology and Biostatistics, Department of Veterinary Medicine, Freie Universität Berlin, 14163 Berlin, Germany; 7 Department of Internal Medicine, RWTH Aachen University Hospital, 52074 Aachen, Germany

**Keywords:** antimicrobial resistance, biocide susceptibility, *Escherichia coli*, one health

## Abstract

Phenotypic susceptibility testing of *Escherichia* (*E.*) *coli* is an essential tool to gain a better understanding of the potential impact of biocide selection pressure on antimicrobial resistance. We, therefore, determined the biocide and antimicrobial susceptibility of 216 extended-spectrum β-lactamase-producing (ESBL) and 177 non-ESBL *E. coli* isolated from swine feces, pork meat, voluntary donors and inpatients and evaluated associations between their susceptibilities. Minimum inhibitory concentrations (MICs) and minimum bactericidal concentrations (MBCs) of benzalkonium chloride, chlorhexidine digluconate (CHG), chlorocresol (PCMC), glutaraldehyde (GDA), isopropanol (IPA), octenidine dihydrochloride and sodium hypochlorite (NaOCl) showed unimodal distributions, indicating the absence of bacterial adaptation to biocides due to the acquisition of resistance mechanisms. Although MIC_95_ and MBC_95_ did not vary more than one doubling dilution step between isolates of porcine and human origin, significant differences in MIC and/or MBC distributions were identified for GDA, CHG, IPA, PCMC and NaOCl. Comparing non-ESBL and ESBL *E. coli*, significantly different MIC and/or MBC distributions were found for PCMC, CHG and GDA. Antimicrobial susceptibility testing revealed the highest frequency of resistant *E. coli* in the subpopulation isolated from inpatients. We observed significant but weakly positive correlations between biocide MICs and/or MBCs and antimicrobial MICs. In summary, our data indicate a rather moderate effect of biocide use on the susceptibility of *E. coli* to biocides and antimicrobials.

## 1. Introduction

Biocides have been applied for decades as disinfectants, antiseptics and preservatives in healthcare settings and along the food production chain, and they play a major role in the prevention of zoonotic diseases [1,2]. However, concerns have been raised that the widespread use of biocides may contribute to the development and spread of biocide and antimicrobial resistance [3,4]. This trend may be accelerated by the COVID-19 pandemic [5] and future pandemics that require extensive biocide usage for prevention and control.

Epidemiological studies identified temporal associations between the application of biocidal substances and a decreasing susceptibility of bacteria to these substances of interest [6,7]. Hardy and colleagues demonstrated an association between the introduction and increased usage of the antiseptics chlorhexidine digluconate (CHG) and octenidine dihydrochloride (OCT) in a hospital and a reduced susceptibility of *Staphylococcus (S.) aureus* to both substances [6]. Similarly, Pidot and colleagues [7] showed that the wide use of handwash alcohols in two hospitals was associated with a reduced susceptibility of *Enterococcus faecium* to isopropanol (IPA). In a recent outbreak investigation, we identified carbapenem-resistant *Klebsiella (K.) pneumoniae* isolates with a decreased susceptibility to CHG in an intensive care unit where patients were routinely washed with CHG to decrease the rate of catheter-related infections [8]. As a matter of concern, reduced CHG susceptibility was associated with resistance to colistin (COL), which was likely caused by an increased efflux of both substances via the same route. Laboratory adaptation studies provide evidence that bacterial exposure to biocides can lead to resistance and cross-resistance to antimicrobials (reviewed in [3]). Dopcea and colleagues [9] showed that in vitro exposure of clinical *S. aureus* to CHG resulted in a reduced susceptibility to this antiseptic as well as to gentamicin (GEN), penicillin or tetracycline (TET) in several of the investigated strains. Similarly, contact of *Escherichia (E.) coli, Listeria monocytogenes*, *Campylobacter coli* and *Salmonella enterica* with increasing subinhibitory concentrations of the quaternary ammonium compound (QAC) didecyldimethylammonium chloride led to reduced biocide susceptibility and to bacterial antimicrobial resistance (AMR) [10]. AMR is a growing threat to animal and human health and to food safety [11]. AMR is caused by the bacterial mechanisms rendering the drugs used to treat infections less effective [12]. However, numerous studies reported contrasting results on the relevance of biocidal use on the development and spread of AMR. For instance, the absence of bacterial adaptation to biocides in field isolates as well as missing or weak associations between biocide and antimicrobial susceptibility contribute to the controversial discussion of this topic [13,14,15,16,17].

*E. coli* is a gram-negative indicator organism to monitor AMR trends [18,19,20]. The microorganism can persist for varying periods of time in the environment [21]. Persistent colonization of food-processing plants can result in recurrent food contamination [22]. Furthermore, *E. coli* is an important nosocomial pathogen [23,24,25]. Previous studies reported the localization of biocide resistance genes such as the efflux pump encoding genes *qacE∆1, qacF, qacH* and *sugE(p)* on extended-spectrum β-lactamase (ESBL) plasmids in *E. coli* [26,27,28]. The colocalization of biocide and antimicrobial resistance genes on plasmids may provide a selective advantage to ESBL *E. coli* in higher biocide concentrations.

In our study, we investigated the susceptibility of 393 *E. coli* isolates from four different sources, namely primary production (swine feces), food (pork meat), voluntary donors and inpatients, to seven widely used biocides. The study population included 216 ESBL and 177 non-ESBL *E. coli*. According to their origin or ESBL carriage, we comparatively analyzed the minimum inhibitory concentration (MIC) and minimum bactericidal concentration (MBC) data of *E. coli* to identify characteristics potentially specific to the different subpopulations. Furthermore, we determined their bacterial susceptibility to 15 antimicrobials and assessed associations between reduced biocide susceptibility and antimicrobial resistance.

## 2. Results

### 2.1. Biocide Susceptibility

MICs and MBCs of glutaraldehyde (GDA), CHG, benzalkonium chloride (BAC), OCT, IPA, sodium hypochlorite (NaOCl) and chlorocresol (PCMC) were determined for 393 *E. coli* isolated from swine feces, pork meat, voluntary donors and inpatients (Table 1 and Table 2). Overall, MIC and MBC data showed non-normal, unimodal distributions for all biocides ranging from three to six (MIC data, Table 1) and from four to seven (MBC data, Table 2) dilution steps. The majority of MICs clustered within one or two consecutive concentrations for all biocides tested. The most predominant MICs of GDA, CHG, BAC, OCT, IPA, NaOCl and PCMC were 256–512 mg/L (94%), 1–2 mg/L (91%), 16–32 mg/L (94%), 2 mg/L (96%), 32,768–65,536 mg/L (95%), 256–512 mg/L (97%) and 256–512 mg/L (99%), respectively. The majority of MBCs clustered at equal or slightly higher concentrations than the corresponding MICs, i.e., 512–1024 mg/L for GDA (92%), 1–4 mg/L for CHG (94%), 16–64 mg/L for BAC (98%), 2–4 mg/L for OCT (94%), 65,536–131,072 mg/L for IPA (88%), 512–1024 mg/L for NaOCl (98%) and 512–1024 mg/L for PCMC (99%). Table S1 provides information on the MIC and MBC results for all isolates and substances tested.

The initial comparative analysis of MIC_95_ and MBC_95_ values revealed minor variation. MIC_95_ and MBC_95_ values were the same (MIC_95_ of OCT and PCMC, MBC_95_ of BAC and NaOCl) or differed by only one dilution step between the four *E. coli* subpopulations of different origin (Table 1 and Table 2). Despite the low variability of MIC_95_ and MBC_95_, statistical analysis using the Kruskal–Wallis test revealed significant differences in the MIC and/or MBC distributions for GDA, CHG, IPA, PCMC and NaOCl depending on the source of isolation (Table 1 and Table 2). Subsequently, we identified which of the four subgroups yielded significantly different MIC and/or MBC distributions by applying the post hoc Dunn–Bonferroni test (Table S2a,b). The inpatient isolates exhibited significantly higher GDA MICs and MBCs in comparison to isolates from pork meat and swine feces as well as significantly higher IPA MICs compared to isolates from voluntary donors. Isolates from pork meat showed significantly lower CHG MICs and MBCs compared to the other three subpopulations and significantly higher IPA MICs compared to isolates from swine feces and inpatients. Additionally, MIC_95_ and/or MBC_95_ values differed between the 177 non-ESBL and the 216 ESBL *E. coli* for GDA, BAC, CHG, OCT and PCMC (Table S3a,b). ESBL *E. coli* showed twofold higher MIC_95_ values for GDA and BAC as well as for the CHG MBC_95_ compared to non-ESBL *E. coli*. In contrast, the MBC_95_ of OCT and PCMC was twofold lower in ESBL *E. coli*. Statistically significant differences between these two subgroups were only found for the MIC of PCMC (*p* = 0.003) as well as the MBC of GDA (*p* = 0.040) and CHG (*p* = 0.021).

### 2.2. Antimicrobial Susceptibility

The antimicrobial resistance profiles of 216 ESBL and 177 non-ESBL *E. coli* are summarized in Table 3. AMR results are provided in Table S4. Resistance to ampicillin (AMP), sulfamethoxazole (SME), trimethoprim (TRI) and TET was most frequently observed. Resistance to ciprofloxacin (CIP), nalidixic acid (NAL), azithromycin (AZI), chloramphenicol (CHL) and GEN was less frequent. Low resistance rates were found for COL, meropenem (MER) and tigecycline (TIG).

**Figure 1:**
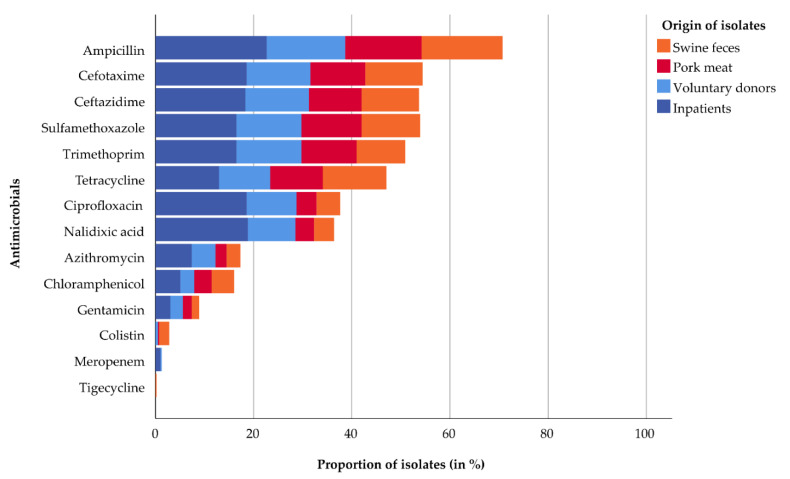
Proportion of antimicrobial-resistant isolates from swine feces (orange), pork meat (red), voluntary donors (light blue) and inpatients (dark blue).

The proportion of isolates resistant to SME, TRI, CIP, NAL, AZI and GEN was higher in the subpopulation isolated from humans than those from swine feces and pork meat. Resistances against TET and CHL were evenly distributed among subgroups (Figure 1). Isolates from inpatients generally displayed the highest frequency of antimicrobial resistance, but they were all susceptible to COL and TIG. We detected only a low resistance rate to MER, with four resistant isolates from inpatients and one from a voluntary donor. COL- and TIG-resistant isolates were rare and were mainly found in swine feces, except for two non-ESBL isolates from voluntary donors and one non-ESBL isolate from pork meat. A total of 251 out of 393 (64%) isolates were resistant to three or more classes of antimicrobials (200 ESBL and 51 non-ESBL *E. coli*), and therefore, they were defined as multidrug-resistant (MDR).

### 2.3. Association between Antimicrobial and Biocide Susceptibility

Significant positive correlations between high MIC and MBC values in biocides and increased MICs in antimicrobials were found in the total *E. coli* population (Table 4) and within each subgroup (Table S5a,b). Correlation coefficients (*r_s_*) ranged from *r_s_* = 0.099 to *r_s_* = 0.280 in the total population and from *r_s_* = 0.201 to *r_s_* = 0.376 within the four origin-related subgroups. In addition, non-ESBL and ESBL *E. coli* isolates were tested separately, and the correlation coefficients ranged from *r_s_* = 0.138 to *r_s_* = 0.307.

In the total *E. coli* population, significant positive correlation coefficients were highest between CHG MBC and NAL MIC (*r_s_* = 0.280), CHG MBC and CIP MIC (*r_s_* = 0.276), CHG MIC and GEN MIC (*r_s_* = 0.228), as well as CHG MIC and MBC and TET MIC (MIC: *r_s_* = 0.229; MBC: *r_s_* = 0.246). Significant positive correlation coefficients within subgroups were highest for BAC MIC and MBC and ceftazidime (CTZ) MIC in isolates from voluntary donors (MIC: *r_s_* = 0.312, MBC: *r_s_* = 0.376) and for CHG MIC and MBC and TET MIC in isolates from pork meat (MIC: *r_s_* = 0.370, MBC: *r_s_* = 0.344). The highest positive and significant correlation coefficient in the non-ESBL and ESBL *E. coli* subpopulations was determined for BAC MBC and CTZ MIC (*r_s_* = 0.307) in non-ESBL isolates. In ESBL isolates, the highest correlation coefficient was observed for CHG MBC and CIP MIC (*r_s_* = 0.259).

## 3. Discussion

In our study, we investigated biocide and antimicrobial susceptibility in a large *E. coli* population (n = 393) including 55% (n = 216) ESBL *E. coli* originating from human (voluntary donors and inpatients) and swine (feces and pork meat) samples to better understand the potential impact of biocide usage on the spread of biocide and antimicrobial-resistant bacteria. We determined distributions of MICs and MBCs for biocidal substances frequently used in animal husbandry, along the food chain and/or in human healthcare settings.

MIC and MBC data showed unimodal distributions, indicating that the investigated *E. coli* did not acquire biocide resistance. OCT and PCMC MIC_95_ values as well as BAC and NaOCl MBC_95_ values were the same for all subgroups. For all other substances tested, we observed slightly different MIC_95_ and/or MBC_95_ values in isolates from inpatients, voluntary donors, swine feces and pork meat. Overall, there was only one doubling dilution step difference in MIC_95_ and/or MBC_95_ values between subgroups. For example, twofold higher MIC_95_ values of CHG were determined for isolates from humans and swine feces (4 mg/L) compared to pork meat isolates (2 mg/L). A similar variability was described by Deus and colleagues, who compared MIC_90_ values of isolates from poultry (1 mg/L) and humans (2 mg/L) [27]. A higher MIC_90_ of CHG at 16 mg/L was reported previously for *E. coli* isolates from hospital patients [14]. Indeed, concerns have been raised about associations between increasing CHG usage in healthcare and an increased MIC of CHG, cross-resistance with antimicrobials and their link to multidrug resistance [29]. In contrast, the comparison of MIC_95_ and MBC_95_ values in our study population indicates rather low variability of biocide susceptibility in different settings independent of the underlying selection pressure. Nevertheless, statistical analysis revealed significant differences in MIC and/or MBC distributions between subpopulations. This might reflect diverse usage patterns of biocidal substances in these four settings. One limitation of our study is that we did not obtain information on the biocides applied or their frequency of use in the sampled areas. Therefore, it is impossible to draw conclusions regarding whether usage patterns may influence biocide susceptibility in *E. coli*. Nonetheless, according to our data, even high selection pressure due to frequent application of disinfectants, antiseptics and antimicrobials (e.g., *E. coli* of hospitalized patients compared to isolates from voluntary donors) seems to have a rather moderate impact on the development of reduced susceptibility to tested substances in *E. coli*. However, single isolates showed considerably high MIC and/or MBC values.

The MIC ranges observed for BAC, CHG, PCMC, NaOCl and GDA in our study were consistent with those of previous studies that investigated *E. coli* isolates from livestock [15,30,31,32], meat [26] and human samples [14,27]. Hitherto, interpretative criteria to distinguish biocide resistant from susceptible *E. coli* isolates are missing, impeding the assessment of biocide susceptibility data [33]. Tentative epidemiological cut-offs (ECOFFs) based on MIC_90_, MIC_95_ or MIC_99.9_ values of clinically relevant microorganisms including *E. coli* have been proposed [14,15,27]. Morrissey and colleagues defined ECOFFs for CHG (MIC_99.9_ = 64 mg/L, MBC_99.9_ > 64 mg/L), BAC (MIC_99.9_ = 64 mg/L, MBC_99.9_ = 128 mg/L) and NaOCl (MIC_99.9_ = 8.2 g/L, MBC_99.9_ = 16.4 g/L) considering 368 *E. coli* isolates collected between 1998 and 2011 [14]. The results obtained in our study for CHG and NaOCl were below these reported tentative ECOFFs, whereas 22 isolates yielded BAC MIC or MBC values above the published thresholds. The observation of similar susceptibilities to CHG, BAC and NaOCl in *E. coli* isolated from various environments geographically and temporally widely dispersed indicates a lack of adaptation to tested substances in the recent past. However, comparing MIC and MBC values across studies remains a challenge because experimental standards for biocide susceptibility testing (BST) are still missing. Therefore, harmonized experiments and standardized methods are essential. Despite the recent development of quality control (QC) ranges for BST [34], standardized ECOFFs or specific breakpoints to define isolates with a reduced susceptibility to biocides still lag behind.

To determine putative associations between an ESBL phenotype and reduced biocide susceptibility, we comparatively analyzed the MIC and MBC distributions of ESBL and non-ESBL *E. coli*. Susceptibility to BAC, IPA, NaOCl, PCMC and OCT was comparable for ESBL and non-ESBL *E. coli*, indicating that ESBL-carriage does not benefit survival during contact with these substances. Even though the MIC_95_ and MBC_95_ values of GDA, CHG and PCMC only differed in one doubling dilution step between both groups, statistically significant differences were observed in GDA and CHG MBC distributions as well as in PCMC MIC distributions. Although GDA and CHG MBCs of ESBL *E. coli* shifted towards higher values compared to the non-ESBL subpopulation, increased PCMC MICs were found in the non-ESBL subset. In our study, we did not observe higher MIC values for the quaternary ammonium compound BAC in ESBL *E. coli*, as previously described [27]. The association observed between the ESBL phenotype and decreased susceptibility to GDA and CHG might be explained by the genetic relatedness of resistance mechanisms.

Interpretation of antimicrobial susceptibility testing data using ECOFFs is a powerful tool for the surveillance of antimicrobial resistance [35]. Further, ECOFFs allow for comparing the development of antimicrobial resistance in bacteria irrespective of their origin [36]. Accordingly, we comparatively analyzed AST data of our study population based on ECOFFs [35]. To enable comparison of AMR in *E. coli* of human origin (inpatients and voluntary donors) with reports from national and international surveillance systems, we considered clinical breakpoints [37] instead of ECOFFs.

Overall, 64% of the tested isolates (57% from healthcare settings and 43% from swine) were resistant to three or more classes of antimicrobials and were thereby classified as MDR. MDR *E. coli* have been widely reported in livestock [15,38,39], food [40] and nosocomial infections [41,42,43,44,45]. As expected, the highest resistance rates to most of the substances, including AMP, SME, TRI, CIP, NAL and AZI, were found in isolates from hospital patients. Non-ESBL isolates from inpatients also showed higher resistance rates to the listed antimicrobials compared to non-ESBL isolates from other subpopulations. In accordance with this, resistance of *E. coli* to AMP, CIP and TRI has been frequently documented in the German antimicrobial resistance surveillance database (ARS) [46]. In our study population, we identified isolates bearing MER resistance, whereas this resistance has not been documented in ARS in the last five years. However, the MER resistance rates found in our study are consistent with previous results [47]. A low number of isolates were resistant to last-resort antimicrobials such as COL and TIG [48]. The World Health Organization recommends the restricted use of both substances exclusively for the treatment of life-threatening infections due to MDR bacteria [48,49]. We identified COL- and TIG-resistant *E. coli* isolates predominantly from swine feces. Interestingly, neither resistance to COL nor to TIG were reported within the German National Zoonoses Monitoring Program for *E. coli* of porcine origin in 2019 [50]. However, COL-resistant *E. coli* have been isolated from German swine farms in the recent past [51]. TIG resistance in *E. coli* from swine has been previously reported from the United Kingdom, South Africa and Thailand [52,53,54]. When comparing *E. coli* AMR data from swine feces and pork meat sampled in our study with reporting data from the German National Zoonoses Monitoring Program as well as with human data with resistance rates documented in ARS, the high number of ESBL *E. coli* included in our study group has to be considered (ranging from 44 to 74% in the four subpopulations). Accordingly, resistance rates were generally higher in the isolates included in our study.

Cross-resistance and co-resistance are discussed as potential drivers for biocide-induced emergence and the spread of antimicrobial resistance [55,56,57,58]. Cross-resistant bacteria have developed one specific mechanism enabling survival at higher concentrations of various antibacterial substances, e.g., biocides and antimicrobials [55]. Examples are the cross-resistance of *K. pneumoniae* to COL and CHG [8,59] and the cross-resistance of *Acinetobacter baumannii* to multiple antimicrobials and triclosan [60]. Co-resistance describes the colocalization of two or more resistance genes encoding for independent resistance mechanisms, which are transferred in a single event and expressed jointly in a new bacterial cell [55,61]. Roedel and colleagues [15] described the colocalization of QAC resistance determinants and antimicrobial resistance genes on mobile genetic elements. Associations between increased biocide and antimicrobial MICs are indicators for co-selection due to cross- or co-resistance. Correlation analysis has often been used to investigate such associations [15,62,63]. In our study, several significant positive correlations between tested biocidal substances and antimicrobials were observed, which were all classified as weak [62]. Hence, our phenotypic data do not provide strong evidence that biocide and antimicrobial resistance are linked. Biocides act against bacterial cells at multiple target sites [55]. Accordingly, different mechanisms such as efflux, degradation and impermeability or even their synergistic action may enable bacteria to survive higher concentrations of a specific biocide. The diversity of bacterial protection mechanisms might be one possible explanation for the weak correlations we observed. Further, it has been shown that prolonged bacterial exposure to low biocide concentrations may result in AMR development, which is not accompanied by a stable decrease in biocide susceptibility [64,65]. Because this pathway to antimicrobial resistance induced by biocides is unrelated to changes in biocide MICs, it cannot be detected with the approach of our study.

In conclusion, our data lack the clear evidence that *E. coli* field isolates have adapted to tested biocidal substances in the recent past. Even though we found statistically significant differences for MIC and MBC distributions between subgroups, the low variability of phenotypic susceptibility data suggests a rather low impact of niche-specific selection pressure on the adaptation of *E. coli* to biocides. Whether the identified correlations between biocide and antimicrobial susceptibility are related to co- or cross-resistance needs to be elucidated in mechanistic analyses.

## 4. Materials and Methods

### 4.1. E. coli Isolates

In total, 393 *E. coli* strains were investigated in our study, including 216 ESBL and 177 non-ESBL phenotypes. *E. coli* isolated from humans originated from the strain collection of the Institute of Infectious Diseases and Infection Control of Jena University Hospital. These isolates were obtained from previous research projects (see section Institutional Review Board Statement). Specifically, rectal swabs were collected from two groups: 96 voluntary donors in 2018 and 98 colonized hospital patients (inpatients) between 2014 and 2017. Isolates of porcine origin comprised 100 *E. coli* from the strain collection of the Institute of Microbiology and Epizootics isolated from swine fecal samples collected at 15 German swine farms in 2016 and 99 pork meat isolates from the National Reference Laboratory for Antimicrobial Resistance located at the German Federal Institute for Risk Assessment (BfR). *E. coli* from pork meat were obtained from the German National Zoonoses Monitoring Program in 2015 and 2017, following the requirements of commission implementing decision (CID) 2013/652/EU. The ESBL phenotypes of all isolates were determined in their respective diagnostic laboratories using either VITEK^®^-2 (bioMérieux Inc., Durham, NC, USA) testing (VITEK^®^-2 software release 8.01) with the AST-GN69 card or using the ESBL confirmatory test according to the Clinical and Laboratory Standards Institute (CLSI) recommendations. Isolates were stored in glycerol stocks and were grown on Mueller–Hinton (MH) agar (Thermo Fisher Diagnostics GmbH Microbiology, Wesel, Germany) overnight at 37 °C before use.

### 4.2. Biocides

The susceptibility of *E. coli* to seven biocides frequently added to antiseptics and disinfectants applied in healthcare and food production was tested, namely GDA (Carl Roth, Karlsruhe, Germany), CHG (Sigma Aldrich, Steinheim, Germany), BAC (Sigma Aldrich), OCT (Thermo Fisher Scientific, Schwerte, Germany), IPA (Carl Roth), NaOCl (AppliChem, Darmstadt, Germany) and PCMC (Sigma Aldrich). The concentrated chemical solutions were freshly diluted prior to the experiments with standardized hard water as defined in EN 1276. Serial twofold dilutions were prepared in 96-well plates within the following concentration ranges: 8192 to 64 mg/L of GDA, 64 to 0.5 mg/L of CHG, 256 to 2 mg/L of BAC, 32 to 0.25 mg/L of OCT, 262,144 to 2048 mg/L of IPA, 4096 to 64 mg/L of NaOCl and 8192 to 64 mg/L of PCMC.

### 4.3. Biocide Susceptibility Testing

The MICs and MBCs of all isolates to each biocide were determined via broth microdilution as previously reported [15]. Briefly, overnight cultures grown on Mueller–Hinton agar (MHA, Mast Diagnostica GmbH, Reinfeld, Germany) were adjusted to 10^6^ colony-forming units (CFU) per milliliter of double-concentrated Mueller–Hinton broth (MHB, Mast Diagnostica GmbH). In a 96-well microtiter plate (Greiner Bio One, Frickenhausen, Germany), 50 μL of this bacterial solution was mixed with 50 μL of the twofold-concentrated biocide. The plates were incubated at 37 °C for 20 ± 2 h. Optical density (OD_595 nm_) was measured after 5 s of shaking using an iMark Microplate Absorbance Reader (Bio-Rad, Feldkirchen, Germany). Bacterial growth was compared to a negative control (microtiter plate containing biocide solution and MHB), and ΔOD_595 nm_ = 0.08 was set as the cut-off. The MIC was defined as the lowest concentration of a biocide at which no growth was observed.

For MBC determination, 20 μL each from those wells without detectable bacterial growth and those with the lowest concentration of biocides at which bacterial growth was only just observed were added to 180 μL of Dey–Engley neutralizing broth solution (Sigma Aldrich) to quench biocidal effects. After a short incubation period (5 min), 25 μL were plated on MHA in duplicates and incubated at 37 °C for 24 h. Dey–Engley neutralizing broth solution was prepared according to the manufacturer’s recommendations. Neutralizer efficacy and toxicity were assessed prior to MIC and MBC testing according to the process of Roedel and colleagues [15]. The MBC was defined as the lowest concentration of the biocide at which no bacterial colonies on MHA were observed. The reference strain *E. coli* ATCC 25922 was used as an internal quality control in both MIC and MBC tests and showed comparable results throughout the experiments. To evaluate the reproducibility of the MIC and MBC tests, both methods were performed in two biological replicates on separate days for a subset of 43 *E. coli* (>10% of the study population) and all biocides. MIC or MBC variations of one dilution step between the two experiments were accepted. The lower concentration was defined as MIC or MBC. In case of higher variations, we repeated the test for a third time, and the median was considered to be the final MIC or MBC (data not shown). The lowest biocide concentration inhibiting growth of 95% of the bacterial population was designated as MIC_95_, and the lowest lethal concentration killing 95% of the bacterial population was designated as MBC_95_.

### 4.4. Antimicrobial Susceptibility Testing

The susceptibility of *E. coli* to antimicrobials was determined via broth microdilution following CLSI guidelines [66] using the Sensititre system with EUVSEC and EUVSEC3 plates (Thermo Fisher Scientific) in concordance with the decisions 2013/652/EU and 2020/1729/EU of the European Union. We analyzed antimicrobial resistance to a variety of substances, namely: AMP, cefotaxime, CTZ, MER, NAL, CIP, GEN, TET, TIG, COL, SME, TRI, CHL and AZI. We defined resistance using ECOFFs according to the European Committee for Antimicrobial Susceptibility Testing (EUCAST) [35] and the decision CID 2020/1729/EU of the European Union.

### 4.5. Statistical Analysis

Biocide MIC and MBC data were tested for normal distribution using the Kolmogorov–Smirnov test. Comparison of MIC and MBC values between isolates of human and porcine origin (four subgroups) was performed using the Kruskal–Wallis test and the post hoc Dunn–Bonferroni test. The Mann–Whitney test was applied to evaluate differences between non-ESBL and ESBL *E. coli*. Differences were considered significant at *p*-values < 0.05. Correlations between biocide susceptibility and antimicrobial resistance were evaluated by calculating Spearman’s rank coefficients (*r_s_*). Positive correlations were defined as weak (0.1 ≤ *r_s_* < 0.4), moderate (0.4 ≤ *r_s_* < 0.7) or strong (0.7 ≤ *r_s_* < 1) [62], and significance was considered at *p*-values < 0.05. All statistical tests were carried out with SPSS (IBM SPSS Statistics, Version 21, IBM Corp., Armonk, NY, USA).

## Figures and Tables

**Table 1:** Minimum inhibitory concentrations of biocides in *E. coli* of human and porcine origin.

Biocide	Origin of Isolates	Number of Isolates with MIC Values (mg/L) of																					MIC_95_	*p*-Value
		0.25	0.5	1	2	4	8	16	32	64	128	256	512	1024	2048	4096	8192	16,384	32,768	65,536	131,072	262,144		
**GDA**	Swine feces									-	-	18	81	1	-	-	-						512	0.002
	Pork meat									-	-	15	82	2	-	-	-						512	
	Voluntary donors									-	-	16	67	13	-	-	-						1024	
	Inpatients									-	-	3	88	6	1	-	-						1024	
**CHG**	Swine feces		-	12	79	9	-	-	-	-													4	<0.0001
	Pork meat		7	50	39	2	1	-	-	-													2	
	Voluntary donors		-	7	77	9	1	2	-	-													4	
	Inpatients		-	11	81	3	3	-	-	-													4	
**BAC**	Swine feces				-	-	-	30	67	3	-	-											32	0.239
	Pork meat				-	-	-	18	81	-	-	-											32	
	Voluntary donors				-	-	3	28	62	3	-	-											32	
	Inpatients				-	-	1	29	54	13	1	-											64	
**OCT**	Swine feces	-	-	4	96	-	-	-	-														2	0.088
	Pork meat	-	-	1	96	2	-	-	-														2	
	Voluntary donors	-	-	1	92	3	-	-	-														2	
	Inpatients	-	-	1	94	3	-	-	-														2	
**IPA**	Swine feces														-	-	-	4	54	40	2	-	65,536	0.022
	Pork meat														-	-	-	1	58	35	5	-	131,072	
	Voluntary donors														-	-	-	5	51	39	1	-	65,536	
	Inpatients														-	-	-	1	38	58	1	-	65,536	
**NaOCl**	Swine feces									-	-	15	79	6	-	-							1024	0.016
	Pork meat									-	-	22	76	1	-	-							512	
	Voluntary donors									-	-	2	66	6	-	-							1024	
	Inpatients									-	-	5	93	-	-	-							512	
**PCMC**	Swine feces									-	-	57	42	1	-	-	-						512	0.001
	Pork meat									-	-	81	18	-	-	-	-						512	
	Voluntary donors									-	1	68	27	-	-	-	-						512	
	Inpatients									-	2	73	23	-	-	-	-						512	

GDA = glutaraldehyde; CHG = chlorhexidine digluconate; BAC = benzalkonium chloride; OCT = octenidine dihydrochloride; IPA = isopropanol; NaOCl = sodium hypochlorite; PCMC = chlorocresol. MIC = minimum inhibitory concentration. MIC_95_ = lowest concentration inhibiting growth of 95% of the bacterial population. Kruskal–Wallis test was applied to identify differences between the MIC distributions of subgroups. Differences were considered significant at *p* < 0.05. The grey areas are the test ranges not included in the test panels for the respective substance.

**Table 2:** Minimum bactericidal concentrations of biocides in *E. coli* of human and porcine origin.

Biocide	Origin of Isolates	Number of Isolates with MBC Values (mg/L) of																					MBC_95_	*p*-Value
		0.25	0.5	1	2	4	8	16	32	64	128	256	512	1024	2048	4096	8192	16,384	32,768	65,536	131,072	262,144		
**GDA**	Swine feces									-	-	9	87	4	-	-	-						512	0.004
	Pork meat									-	-	6	90	3	-	-	-						512	
	Voluntary donors									-	-	11	68	16	1	-	-						1024	
	Inpatients									-	-	3	78	16	1	-	-						1024	
CHG	Swine feces		-	10	73	14	2	1	-	-													4	<0.0001
	Pork meat		6	49	37	6	1	-	-	-													4	
	Voluntary donors		-	4	75	12	3	2	-	-													8	
	Inpatients		-	9	57	24	7	-	1	-													8	
**BAC**	Swine feces				-	-	-	13	71	16	-	-											64	0.069
	Pork meat				-	-	-	7	74	16	2	-											64	
	Voluntary donors				-	-	1	22	60	13	-	-											64	
	Inpatients				-	-	1	23	52	20	2	-											64	
**OCT**	Swine feces	-	-	3	82	12	3	-	-														4	0.704
	Pork meat	-	-	-	83	11	5	-	-														8	
	Voluntary donors	-	-	1	82	9	4	-	-														4	
	Inpatients	-	-	-	81	11	6	-	-														8	
**IPA**	Swine feces														-	-	-	-	4	19	73	3	131,072	0.009
	Pork meat														-	-	-	-	4	12	65	18	262,144	
	Voluntary donors														-	-	-	1	3	19	68	5	262,144	
	Inpatients														-	-	-	-	5	18	73	2	131,072	
**NaOCl**	Swine feces									-	-	-	86	13	1	-							1024	0.178
	Pork meat									-	-	-	91	8	-	-							1024	
	Voluntary donors									-	-	5	72	18	1	-							1024	
	Inpatients									-	-	-	92	6	-	-							1024	
**PCMC**	Swine feces									-	-	1	90	9	-	-	-						1024	0.022
	Pork meat									-	-	-	99	-	-	-	-						512	
	Voluntary donors									-	-	2	84	10	-	-	-						1024	
	Inpatients									-	1	2	92	3	-	-	-						512	

GDA = glutaraldehyde; CHG = chlorhexidine digluconate; BAC = benzalkonium chloride; OCT = octenidine dihydrochloride; IPA = isopropanol; NaOCl = sodium hypochlorite; PCMC = chlorocresol. MBC = minimum bactericidal concentration. MBC_95_ = lowest lethal concentration killing 95% of the bacterial population. Kruskal–Wallis test was applied to identify differences between the MBC distributions of subgroups. Differences were considered significant at *p* < 0.05. The grey areas are the test ranges not included in the test panels for the respective substance.

**Table 3:** Frequency of antimicrobial resistance in non-ESBL and ESBL *E. coli* isolates.

Antimicrobials	Swine Feces, n (%)		Pork Meat, n (%)		Voluntary Donors, n (%)		Inpatients, n (%)		Total, n (%)
	ESBL (n = 48)	Non-ESBL (n = 52)	ESBL (n = 44)	Non-ESBL (n = 55)	ESBL (n = 51)	Non-ESBL (n = 45)	ESBL (n = 73)	Non-ESBL (n = 25)	*E. coli* (n = 393)
Ampicillin	48 (100)	17 (33)	44 (100)	17 (31)	51 (100)	12 (27)	73 (100)	16 (64)	278 (71)
Cefotaxime	48 (100)	0	44 (100)	0	51 (100)	0	73 (100)	0	214 (55)
Ceftazidime	48 (100)	0	42 (95)	0	51 (100)	0	72 (99)	0	211 (54)
Meropenem	0	0	0	0	1 (2)	0	4 (5)	0	5 (1)
Nalidixic acid	11 (23)	5 (10)	12 (27)	3 (5)	32 (63)	6 (13)	60 (82)	14 (56)	143 (36)
Ciprofloxacin	12 (25)	7 (13)	13 (30)	3 (5)	33 (65)	7 (16)	59 (81)	14 (56)	148 (38)
Gentamicin	4 (8)	2 (4)	6 (14)	1 (2)	7 (14)	3 (7)	11 (15)	1 (4)	35 (9)
Tetracycline	30 (63)	21 (40)	22 (50)	20 (36)	30 (59)	11 (24)	41 (56)	10 (40)	185 (47)
Tigecycline	1 (2)	0	0	0	0	0	0	0	1 (0.3)
Colistin	7 (15)	1 (2)	0	1 (2)	0	2 (4)	0	0	11 (3)
Sulfamethoxazole	31 (65)	16 (31)	30 (68)	18 (33)	42 (82)	10 (22)	53 (73)	12 (48)	212 (54)
Trimethoprim	28 (58)	11 (21)	26 (59)	18 (33)	43 (84)	9 (20)	52 (71)	13 (52)	200 (51)
Chloramphenicol	13 (27)	5 (10)	5 (11)	9 (16)	8 (16)	3 (7)	16 (22)	4 (16)	63 (16)
Azithromycin	10 (21)	1 (2)	9 (21)	0	16 (31)	3 (7)	24 (33)	5 (20)	68 (17)

ESBL = Extended-spectrum β-lactamase-producing *E. coli*.

**Table 4:** Significant positive correlation coefficients (*r_s_*) between biocides (MIC or MBC) and antimicrobials (MIC) in 393 *E. coli* isolates.

Antimicrobials	Biocides					
	Glutaraldehyde		Chlorhexidine Digluconate		Benzalkonium Chloride	
	MIC	MBC	MIC	MBC	MIC	MBC
Ampicillin	-	-	-	-	0.119 **	0.121 *
Cefotaxime	-	0.100 *	-	0.117 *	-	-
Ceftazidime	-	-	-	-	0.111 *	-
Meropenem	0.127 *	0.110 *	-	-	-	-
Nalidixic acid	0.113 *	-	0.198 **	0.280 **	0.136 **	-
Ciprofloxacin	0.109 *	-	0.208 **	0.276 **	0.144 **	-
Gentamicin	0.164 **	-	0.228 **	0.195 **		-
Tetracycline	-	-	0.229 **	0.246 **	0.100 *	-
Tigecycline	-	-	-	-	-	0.121 *
Sulfamethoxazole	-	-	-	-	0.155 **	0.128 *
Trimethoprim	-	-	-	-	0.144 **	0.155 **
Chloramphenicol	-	-	0.109 *	0.103 *	-	0.099 *
Azithromycin	-	-	0.152 **	0.138 **	0.173 **	0.152**

MIC = minimum inhibitory concentration; MBC = minimum bactericidal concentration; * correlation is significant at the 0.05 level; ** correlation is significant at the 0.01 level.

## Data Availability

All data are presented within the text and in the Supplementary Materials.

## Supplementary Materials

The following supporting information can be downloaded at: https://www.mdpi.com/article/10.3390/antibiotics12050823/s1, Table S1: Biocide susceptibility of 393 *E. coli*; Table S2a: Pairwise comparisons of MIC distributions of *E. coli* of human and porcine origin; Table S2b: Pairwise comparisons of MBC distributions of *E. coli* of human and porcine origin; Table S3a: Minimum inhibitory concentrations of biocides in non-ESBL and ESBL *E. coli* isolates; Table S3b: Minimum bactericidal concentrations of biocides in non-ESBL and ESBL *E. coli* isolates; Table S4: Antimicrobial susceptibility of 393 *E. coli*; Table S5a: Significant positive correlation coefficients (*r_s_*) between biocides and antimicrobials in *E. coli* of human and porcine origin; Table S5b: Significant positive correlation coefficients (*r_s_*) between biocides and antimicrobials in non-ESBL and ESBL *E. coli*.
