# Supplementary material for: Biocide Susceptibility and Antimicrobial Resistance of Escherichia coli Isolated from Swine Feces, Pork Meat and Humans in Germany
Source: Antibiotics (Basel). 2023 Apr 27;12(5):823. doi: 10.3390/antibiotics12050823 (PMC10215396; doi:10.3390/antibiotics12050823)
Supplement: Supplementary file 1 [file antibiotics-12-00823-s001.zip › antibiotics-2359558-supplementary/Table S1.pdf]

Table S1. Biocide susceptibility of 393 *E. coli* .

BfR-ID	Origin of isolates	Resistance	MIC (mg/L)				
			GDA	CHG	BAC	OCT	IPA
18-47-134	Voluntary donor	ESBL	512.0	2.0	32.0	2.0	32768.0
18-47-135	Voluntary donor	ESBL	256.0	2.0	32.0	2.0	65536.0
18-47-136	Voluntary donor	ESBL	512.0	2.0	32.0	2.0	32768.0
18-47-137	Voluntary donor	ESBL	256.0	1.0	32.0	2.0	32768.0
18-47-138	Voluntary donor	ESBL	512.0	2.0	32.0	2.0	65536.0
18-47-139	Voluntary donor	ESBL	512.0	4.0	32.0	2.0	32768.0
18-47-140	Voluntary donor	ESBL	512.0	1.0	32.0	2.0	65536.0
18-47-141	Voluntary donor	ESBL	512.0	2.0	32.0	2.0	32768.0
18-47-142	Voluntary donor	ESBL	512.0	8.0	32.0	2.0	32768.0
18-47-143	Voluntary donor	ESBL	512.0	2.0	32.0	2.0	32768.0
18-47-144	Voluntary donor	ESBL	512.0	2.0	8.0	2.0	32768.0
18-47-150	Voluntary donor	ESBL	512.0	16.0	64.0	2.0	32768.0
18-47-151	Voluntary donor	ESBL	512.0	2.0	32.0	2.0	32768.0
18-47-152	Voluntary donor	ESBL	512.0	2.0	32.0	2.0	65536.0
18-47-154	Voluntary donor	ESBL	512.0	4.0	32.0	2.0	16384.0
18-47-155	Voluntary donor	ESBL	512.0	2.0	32.0	2.0	32768.0
18-47-157	Voluntary donor	non-ESBL	512.0	2.0	32.0	2.0	32768.0
18-47-158	Voluntary donor	non-ESBL	512.0	4.0	32.0	2.0	32768.0
18-47-159	Voluntary donor	non-ESBL	512.0	2.0	32.0	2.0	32768.0
18-47-160	Voluntary donor	non-ESBL	512.0	2.0	32.0	2.0	32768.0
18-47-161	Voluntary donor	non-ESBL	512.0	2.0	32.0	2.0	32768.0
18-47-162	Voluntary donor	non-ESBL	512.0	2.0	16.0	2.0	32768.0
18-47-164	Voluntary donor	non-ESBL	512.0	2.0	32.0	2.0	32768.0
18-47-167	Voluntary donor	non-ESBL	512.0	1.0	32.0	2.0	65536.0
18-47-168	Voluntary donor	non-ESBL	512.0	2.0	16.0	4.0	16384.0
18-47-169	Voluntary donor	non-ESBL	512.0	2.0	8.0	2.0	32768.0
18-47-170	Voluntary donor	non-ESBL	256.0	2.0	32.0	2.0	32768.0
18-47-171	Voluntary donor	non-ESBL	256.0	2.0	32.0	2.0	32768.0
18-47-172	Voluntary donor	non-ESBL	256.0	4.0	16.0	2.0	32768.0
18-47-174	Voluntary donor	ESBL	256.0	1.0	32.0	2.0	32768.0
18-47-176	Voluntary donor	ESBL	512.0	2.0	16.0	2.0	32768.0
18-47-177	Voluntary donor	ESBL	256.0	2.0	32.0	2.0	65536.0
18-47-178	Voluntary donor	ESBL	512.0	2.0	16.0	2.0	32768.0
18-47-179	Voluntary donor	ESBL	512.0	2.0	16.0	2.0	32768.0
18-47-180	Voluntary donor	ESBL	256.0	2.0	64.0	2.0	131072.0
18-47-183	Voluntary donor	non-ESBL	256.0	2.0	32.0	2.0	65536.0
18-47-184	Voluntary donor	non-ESBL	512.0	2.0	16.0	2.0	32768.0
18-47-187	Voluntary donor	non-ESBL	256.0	2.0	32.0	2.0	65536.0
18-47-188	Voluntary donor	non-ESBL	512.0	2.0	16.0	2.0	16384.0
18-47-189	Voluntary donor	non-ESBL	256.0	1.0	32.0	2.0	16384.0
18-47-190	Voluntary donor	non-ESBL	512.0	2.0	8.0	2.0	32768.0
18-47-191	Voluntary donor	non-ESBL	512.0	2.0	16.0	2.0	32768.0
18-47-192	Voluntary donor	non-ESBL	512.0	2.0	16.0	2.0	32768.0
18-47-193	Voluntary donor	non-ESBL	512.0	2.0	16.0	2.0	32768.0
18-47-194	Voluntary donor	non-ESBL	512.0	2.0	16.0	2.0	65536.0
18-47-195	Voluntary donor	non-ESBL	1024.0	2.0	16.0	4.0	65536.0
18-47-196	Voluntary donor	non-ESBL	512.0	2.0	16.0	2.0	65536.0
18-47-197	Voluntary donor	non-ESBL	512.0	2.0	16.0	2.0	65536.0

18-47-199	Voluntary donor	non-ESBL	512.0	2.0	32.0	2.0	65536.0
18-47-202	Voluntary donor	non-ESBL	1024.0	2.0	16.0	2.0	65536.0
18-47-203	Voluntary donor	ESBL	512.0	2.0	32.0	2.0	65536.0
18-47-204	Voluntary donor	ESBL	512.0	2.0	16.0	2.0	32768.0
18-47-208	Voluntary donor	ESBL	512.0	2.0	16.0	4.0	32768.0
18-47-209	Voluntary donor	ESBL	512.0	2.0	16.0	2.0	32768.0
18-47-210	Voluntary donor	ESBL	1024.0	2.0	16.0	2.0	65536.0
18-47-211	Voluntary donor	ESBL	1024.0	2.0	16.0	2.0	32768.0
18-47-213	Voluntary donor	ESBL	1024.0	2.0	16.0	2.0	32768.0
18-47-224	Voluntary donor	non-ESBL	512.0	2.0	16.0	2.0	65536.0
18-47-225	Voluntary donor	non-ESBL	1024.0	2.0	16.0	2.0	32768.0
18-47-226	Voluntary donor	ESBL	1024.0	2.0	32.0	2.0	32768.0
18-47-227	Voluntary donor	ESBL	1024.0	1.0	32.0	1.0	16384.0
18-47-228	Voluntary donor	ESBL	1024.0	2.0	32.0	2.0	32768.0
18-47-229	Inpatient	ESBL	1024.0	2.0	32.0	2.0	32768.0
18-47-230	Voluntary donor	ESBL	512.0	2.0	32.0	2.0	32768.0
18-47-231	Voluntary donor	ESBL	512.0	2.0	32.0	2.0	32768.0
18-47-232	Inpatient	ESBL	512.0	2.0	32.0	2.0	32768.0
18-47-233	Voluntary donor	ESBL	512.0	2.0	32.0	2.0	32768.0
18-47-235	Voluntary donor	ESBL	512.0	2.0	32.0	2.0	32768.0
18-47-239	Voluntary donor	ESBL	512.0	2.0	32.0	2.0	32768.0
18-47-241	Voluntary donor	ESBL	512.0	4.0	32.0	2.0	32768.0
18-47-242	Voluntary donor	ESBL	512.0	2.0	32.0	2.0	32768.0
18-47-244	Voluntary donor	ESBL	256.0	2.0	32.0	2.0	65536.0
18-47-246	Voluntary donor	ESBL	512.0	2.0	32.0	2.0	32768.0
18-47-247	Voluntary donor	ESBL	512.0	2.0	32.0	2.0	32768.0
18-47-248	Voluntary donor	ESBL	512.0	16.0	64.0	2.0	32768.0
18-47-249	Voluntary donor	ESBL	512.0	4.0	32.0	2.0	32768.0
18-47-251	Voluntary donor	ESBL	1024.0	2.0	16.0	2.0	65536.0
18-47-252	Voluntary donor	ESBL	512.0	2.0	32.0	2.0	65536.0
18-47-253	Voluntary donor	ESBL	1024.0	2.0	32.0	2.0	65536.0
18-47-342	Voluntary donor	non-ESBL	512.0	2.0	16.0	2.0	65536.0
18-47-344	Voluntary donor	non-ESBL	1024.0	2.0	32.0	2.0	65536.0
18-47-345	Voluntary donor	non-ESBL	512.0	2.0	32.0	2.0	65536.0
18-47-347	Voluntary donor	non-ESBL	512.0	2.0	32.0	2.0	65536.0
18-47-348	Voluntary donor	non-ESBL	512.0	2.0	32.0	2.0	65536.0
18-47-349	Voluntary donor	non-ESBL	512.0	2.0	32.0	2.0	65536.0
18-47-350	Voluntary donor	non-ESBL	512.0	2.0	32.0	2.0	65536.0
18-47-351	Voluntary donor	non-ESBL	512.0	2.0	16.0	2.0	65536.0
18-47-352	Voluntary donor	non-ESBL	1024.0	2.0	32.0	2.0	65536.0
18-47-353	Voluntary donor	non-ESBL	512.0	2.0	32.0	2.0	65536.0
18-47-358	Voluntary donor	non-ESBL	256.0	2.0	32.0	2.0	65536.0
18-47-359	Voluntary donor	non-ESBL	256.0	4.0	32.0	2.0	65536.0
18-47-360	Voluntary donor	non-ESBL	512.0	4.0	32.0	2.0	65536.0
18-47-362	Voluntary donor	non-ESBL	512.0	4.0	32.0	2.0	65536.0
18-47-363	Voluntary donor	non-ESBL	512.0	2.0	32.0	2.0	65536.0
18-47-364	Voluntary donor	non-ESBL	512.0	2.0	16.0	2.0	65536.0
18-47-369	Voluntary donor	ESBL	512.0	2.0	32.0	2.0	32768.0
18-47-377	Voluntary donor	ESBL	256.0	2.0	32.0	2.0	65536.0
18-47-378	Voluntary donor	ESBL	256.0	1.0	32.0	2.0	65536.0
20-47-10	Swine feces	ESBL	512.0	2.0	32.0	2.0	65536.0

20-47-100	Pork meat	non-ESBL	512.0	1.0	32.0	2.0	65536.0
20-47-101	Pork meat	non-ESBL	512.0	1.0	32.0	2.0	65536.0
20-47-102	Pork meat	ESBL	512.0	1.0	32.0	2.0	65536.0
20-47-103	Pork meat	ESBL	512.0	1.0	32.0	2.0	65536.0
20-47-104	Pork meat	ESBL	256.0	1.0	16.0	2.0	131072.0
20-47-105	Pork meat	non-ESBL	512.0	1.0	16.0	2.0	65536.0
20-47-106	Pork meat	non-ESBL	512.0	1.0	16.0	2.0	65536.0
20-47-107	Pork meat	ESBL	512.0	1.0	32.0	2.0	32768.0
20-47-108	Pork meat	ESBL	512.0	1.0	32.0	2.0	65536.0
20-47-109	Pork meat	non-ESBL	512.0	1.0	16.0	2.0	65536.0
20-47-11	Swine feces	ESBL	512.0	1.0	16.0	2.0	65536.0
20-47-110	Pork meat	ESBL	512.0	1.0	32.0	2.0	65536.0
20-47-111	Pork meat	ESBL	512.0	1.0	32.0	2.0	65536.0
20-47-112	Pork meat	ESBL	512.0	1.0	32.0	2.0	65536.0
20-47-113	Pork meat	ESBL	512.0	2.0	32.0	2.0	65536.0
20-47-114	Pork meat	ESBL	512.0	1.0	32.0	2.0	65536.0
20-47-115	Pork meat	non-ESBL	512.0	1.0	32.0	2.0	131072.0
20-47-116	Pork meat	non-ESBL	512.0	1.0	32.0	2.0	65536.0
20-47-117	Pork meat	non-ESBL	512.0	1.0	32.0	2.0	65536.0
20-47-119	Pork meat	non-ESBL	512.0	1.0	32.0	2.0	32768.0
20-47-12	Swine feces	ESBL	512.0	2.0	32.0	2.0	65536.0
20-47-120	Pork meat	ESBL	512.0	2.0	32.0	2.0	32768.0
20-47-121	Pork meat	ESBL	512.0	2.0	16.0	2.0	32768.0
20-47-122	Pork meat	ESBL	512.0	1.0	32.0	2.0	32768.0
20-47-123	Pork meat	non-ESBL	256.0	1.0	16.0	2.0	32768.0
20-47-124	Pork meat	ESBL	512.0	0.5	32.0	2.0	32768.0
20-47-125	Pork meat	non-ESBL	256.0	1.0	32.0	2.0	32768.0
20-47-126	Pork meat	non-ESBL	512.0	1.0	32.0	2.0	32768.0
20-47-127	Pork meat	non-ESBL	512.0	0.5	32.0	2.0	65536.0
20-47-128	Pork meat	non-ESBL	512.0	0.5	32.0	2.0	65536.0
20-47-129	Pork meat	non-ESBL	512.0	0.5	32.0	2.0	65536.0
20-47-13	Swine feces	ESBL	512.0	2.0	32.0	2.0	65536.0
20-47-130	Pork meat	ESBL	512.0	0.5	16.0	2.0	32768.0
20-47-131	Pork meat	non-ESBL	512.0	4.0	32.0	4.0	32768.0
20-47-132	Pork meat	non-ESBL	512.0	0.5	32.0	2.0	32768.0
20-47-133	Pork meat	non-ESBL	512.0	2.0	32.0	2.0	32768.0
20-47-134	Pork meat	non-ESBL	512.0	1.0	32.0	2.0	32768.0
20-47-135	Pork meat	non-ESBL	512.0	1.0	32.0	4.0	65536.0
20-47-136	Pork meat	ESBL	512.0	1.0	32.0	2.0	65536.0
20-47-137	Pork meat	non-ESBL	512.0	1.0	32.0	2.0	65536.0
20-47-138	Pork meat	non-ESBL	512.0	1.0	32.0	2.0	32768.0
20-47-139	Pork meat	ESBL	512.0	1.0	32.0	2.0	65536.0
20-47-140	Pork meat	ESBL	512.0	1.0	32.0	2.0	65536.0
20-47-141	Pork meat	ESBL	512.0	1.0	32.0	2.0	32768.0
20-47-142	Pork meat	non-ESBL	512.0	1.0	16.0	1.0	65536.0
20-47-143	Pork meat	non-ESBL	512.0	1.0	32.0	2.0	32768.0
20-47-144	Pork meat	non-ESBL	512.0	1.0	32.0	2.0	65536.0
20-47-145	Pork meat	non-ESBL	256.0	2.0	32.0	2.0	32768.0
20-47-146	Pork meat	non-ESBL	512.0	1.0	32.0	2.0	32768.0
20-47-147	Pork meat	non-ESBL	256.0	2.0	32.0	2.0	32768.0
20-47-148	Pork meat	non-ESBL	512.0	1.0	32.0	2.0	32768.0

20-47-149	Pork meat	non-ESBL	512.0	1.0	32.0	2.0	131072.0
20-47-150	Pork meat	non-ESBL	512.0	1.0	32.0	2.0	65536.0
20-47-151	Pork meat	ESBL	512.0	0.5	16.0	2.0	65536.0
20-47-152	Pork meat	non-ESBL	512.0	2.0	16.0	2.0	32768.0
20-47-153	Pork meat	non-ESBL	256.0	1.0	32.0	2.0	65536.0
20-47-154	Pork meat	non-ESBL	512.0	1.0	32.0	2.0	32768.0
20-47-155	Pork meat	non-ESBL	512.0	2.0	32.0	2.0	32768.0
20-47-156	Pork meat	ESBL	512.0	2.0	16.0	2.0	65536.0
20-47-157	Pork meat	ESBL	256.0	4.0	32.0	2.0	65536.0
20-47-158	Pork meat	non-ESBL	512.0	2.0	32.0	2.0	32768.0
20-47-159	Pork meat	non-ESBL	512.0	2.0	32.0	2.0	32768.0
20-47-16	Swine feces	ESBL	512.0	2.0	32.0	2.0	65536.0
20-47-160	Pork meat	non-ESBL	512.0	2.0	32.0	2.0	131072.0
20-47-161	Pork meat	ESBL	512.0	2.0	32.0	2.0	32768.0
20-47-162	Pork meat	ESBL	512.0	2.0	32.0	2.0	32768.0
20-47-163	Pork meat	non-ESBL	512.0	2.0	32.0	2.0	32768.0
20-47-164	Pork meat	ESBL	512.0	2.0	32.0	2.0	32768.0
20-47-165	Pork meat	ESBL	1024.0	2.0	16.0	2.0	32768.0
20-47-166	Pork meat	ESBL	512.0	2.0	32.0	2.0	32768.0
20-47-167	Pork meat	non-ESBL	1024.0	2.0	32.0	2.0	32768.0
20-47-168	Pork meat	non-ESBL	256.0	2.0	16.0	2.0	65536.0
20-47-169	Pork meat	ESBL	512.0	2.0	32.0	2.0	32768.0
20-47-170	Pork meat	ESBL	512.0	2.0	32.0	2.0	32768.0
20-47-171	Pork meat	ESBL	512.0	2.0	32.0	2.0	32768.0
20-47-172	Pork meat	ESBL	512.0	2.0	32.0	2.0	16384.0
20-47-173	Pork meat	ESBL	512.0	2.0	32.0	2.0	32768.0
20-47-174	Pork meat	ESBL	256.0	2.0	16.0	2.0	32768.0
20-47-175	Pork meat	ESBL	256.0	2.0	16.0	2.0	65536.0
20-47-176	Pork meat	ESBL	512.0	2.0	32.0	2.0	32768.0
20-47-177	Pork meat	ESBL	256.0	2.0	32.0	2.0	131072.0
20-47-178	Pork meat	ESBL	512.0	2.0	32.0	2.0	32768.0
20-47-179	Pork meat	ESBL	512.0	2.0	32.0	2.0	32768.0
20-47-18	Swine feces	ESBL	512.0	2.0	32.0	2.0	65536.0
20-47-180	Pork meat	ESBL	512.0	2.0	32.0	2.0	32768.0
20-47-181	Pork meat	non-ESBL	256.0	2.0	32.0	2.0	32768.0
20-47-182	Pork meat	non-ESBL	512.0	2.0	32.0	2.0	32768.0
20-47-183	Swine feces	non-ESBL	512.0	2.0	32.0	2.0	32768.0
20-47-184	Swine feces	non-ESBL	512.0	2.0	16.0	2.0	32768.0
20-47-185	Swine feces	non-ESBL	512.0	2.0	32.0	2.0	32768.0
20-47-186	Swine feces	non-ESBL	512.0	4.0	32.0	2.0	32768.0
20-47-187	Swine feces	non-ESBL	256.0	2.0	32.0	2.0	32768.0
20-47-188	Swine feces	non-ESBL	256.0	2.0	32.0	2.0	32768.0
20-47-189	Swine feces	non-ESBL	512.0	2.0	32.0	2.0	32768.0
20-47-19	Swine feces	ESBL	512.0	2.0	32.0	2.0	32768.0
20-47-190	Swine feces	non-ESBL	256.0	2.0	32.0	2.0	32768.0
20-47-191	Swine feces	non-ESBL	256.0	2.0	32.0	1.0	65536.0
20-47-192	Swine feces	non-ESBL	256.0	2.0	32.0	2.0	131072.0
20-47-193	Swine feces	non-ESBL	512.0	2.0	32.0	2.0	32768.0
20-47-194	Swine feces	non-ESBL	512.0	4.0	16.0	2.0	32768.0
20-47-195	Swine feces	non-ESBL	512.0	2.0	16.0	2.0	32768.0
20-47-197	Swine feces	non-ESBL	512.0	2.0	32.0	2.0	32768.0

20-47-198	Swine feces	non-ESBL	512.0	2.0	32.0	2.0	32768.0
20-47-199	Swine feces	non-ESBL	512.0	4.0	64.0	2.0	65536.0
20-47-2	Swine feces	ESBL	1024.0	2.0	64.0	2.0	65536.0
20-47-20	Swine feces	ESBL	512.0	2.0	32.0	2.0	65536.0
20-47-200	Swine feces	non-ESBL	256.0	2.0	32.0	2.0	32768.0
20-47-202	Swine feces	non-ESBL	512.0	2.0	32.0	2.0	65536.0
20-47-203	Swine feces	non-ESBL	512.0	2.0	16.0	2.0	32768.0
20-47-204	Swine feces	non-ESBL	512.0	2.0	32.0	2.0	65536.0
20-47-205	Swine feces	non-ESBL	512.0	2.0	16.0	2.0	32768.0
20-47-206	Swine feces	non-ESBL	512.0	2.0	32.0	2.0	65536.0
20-47-207	Swine feces	non-ESBL	512.0	2.0	32.0	2.0	65536.0
20-47-208	Swine feces	non-ESBL	512.0	2.0	32.0	2.0	65536.0
20-47-209	Swine feces	non-ESBL	512.0	2.0	32.0	2.0	65536.0
20-47-21	Swine feces	ESBL	512.0	4.0	16.0	2.0	32768.0
20-47-210	Swine feces	non-ESBL	512.0	2.0	16.0	2.0	65536.0
20-47-211	Swine feces	non-ESBL	512.0	2.0	32.0	2.0	65536.0
20-47-212	Swine feces	non-ESBL	512.0	2.0	32.0	2.0	65536.0
20-47-213	Swine feces	non-ESBL	512.0	2.0	32.0	2.0	32768.0
20-47-214	Swine feces	non-ESBL	512.0	2.0	16.0	2.0	131072.0
20-47-215	Swine feces	non-ESBL	512.0	2.0	16.0	2.0	65536.0
20-47-216	Swine feces	non-ESBL	512.0	2.0	64.0	2.0	65536.0
20-47-217	Swine feces	non-ESBL	512.0	2.0	32.0	2.0	65536.0
20-47-219	Swine feces	non-ESBL	512.0	2.0	32.0	2.0	65536.0
20-47-220	Swine feces	non-ESBL	512.0	2.0	32.0	2.0	32768.0
20-47-222	Swine feces	non-ESBL	512.0	2.0	32.0	2.0	32768.0
20-47-223	Swine feces	non-ESBL	512.0	2.0	32.0	2.0	32768.0
20-47-224	Swine feces	non-ESBL	256.0	1.0	32.0	2.0	65536.0
20-47-225	Swine feces	non-ESBL	256.0	2.0	32.0	2.0	32768.0
20-47-226	Swine feces	non-ESBL	512.0	2.0	32.0	2.0	32768.0
20-47-227	Swine feces	non-ESBL	256.0	2.0	32.0	2.0	32768.0
20-47-228	Swine feces	non-ESBL	512.0	2.0	32.0	2.0	65536.0
20-47-229	Swine feces	non-ESBL	512.0	2.0	32.0	2.0	32768.0
20-47-23	Swine feces	ESBL	512.0	2.0	16.0	2.0	32768.0
20-47-230	Swine feces	non-ESBL	512.0	2.0	32.0	2.0	32768.0
20-47-231	Swine feces	non-ESBL	512.0	2.0	32.0	2.0	32768.0
20-47-232	Swine feces	non-ESBL	512.0	2.0	32.0	2.0	65536.0
20-47-233	Swine feces	non-ESBL	512.0	2.0	32.0	2.0	32768.0
20-47-24	Swine feces	ESBL	512.0	2.0	16.0	2.0	16384.0
20-47-25	Swine feces	ESBL	512.0	2.0	32.0	2.0	32768.0
20-47-27	Swine feces	ESBL	512.0	4.0	32.0	2.0	65536.0
20-47-28	Swine feces	ESBL	512.0	2.0	32.0	2.0	65536.0
20-47-29	Swine feces	ESBL	512.0	2.0	16.0	1.0	65536.0
20-47-30	Swine feces	ESBL	256.0	2.0	16.0	2.0	32768.0
20-47-31	Swine feces	ESBL	512.0	2.0	16.0	2.0	32768.0
20-47-32	Swine feces	ESBL	512.0	2.0	16.0	2.0	32768.0
20-47-33	Swine feces	ESBL	512.0	2.0	32.0	2.0	32768.0
20-47-34	Swine feces	ESBL	512.0	2.0	32.0	2.0	32768.0
20-47-35	Swine feces	ESBL	512.0	2.0	16.0	1.0	32768.0
20-47-36	Swine feces	ESBL	512.0	2.0	16.0	1.0	32768.0
20-47-37	Swine feces	ESBL	256.0	2.0	16.0	2.0	32768.0
20-47-38	Swine feces	ESBL	512.0	2.0	16.0	2.0	16384.0

20-47-4	Swine feces	ESBL	512.0	4.0	32.0	2.0	65536.0
20-47-40	Swine feces	ESBL	512.0	2.0	16.0	2.0	16384.0
20-47-41	Swine feces	ESBL	512.0	2.0	32.0	2.0	32768.0
20-47-42	Swine feces	ESBL	512.0	4.0	16.0	2.0	32768.0
20-47-43	Swine feces	ESBL	256.0	4.0	16.0	2.0	32768.0
20-47-44	Swine feces	ESBL	256.0	4.0	32.0	2.0	32768.0
20-47-45	Swine feces	ESBL	256.0	2.0	16.0	2.0	16384.0
20-47-46	Swine feces	ESBL	512.0	1.0	16.0	2.0	32768.0
20-47-47	Swine feces	ESBL	512.0	2.0	32.0	2.0	65536.0
20-47-48	Swine feces	ESBL	512.0	1.0	32.0	2.0	32768.0
20-47-49	Swine feces	ESBL	512.0	1.0	16.0	2.0	32768.0
20-47-5	Swine feces	ESBL	512.0	2.0	32.0	2.0	65536.0
20-47-50	Swine feces	ESBL	512.0	1.0	32.0	2.0	32768.0
20-47-51	Swine feces	ESBL	512.0	1.0	32.0	2.0	32768.0
20-47-52	Swine feces	ESBL	512.0	1.0	32.0	2.0	32768.0
20-47-53	Swine feces	ESBL	512.0	1.0	16.0	2.0	32768.0
20-47-55	Swine feces	ESBL	512.0	2.0	32.0	2.0	32768.0
20-47-56	Swine feces	ESBL	512.0	1.0	16.0	2.0	32768.0
20-47-58	Swine feces	ESBL	256.0	2.0	32.0	2.0	65536.0
20-47-59	Swine feces	ESBL	256.0	1.0	16.0	2.0	32768.0
20-47-6	Swine feces	ESBL	256.0	2.0	32.0	2.0	65536.0
20-47-60	Swine feces	ESBL	256.0	2.0	32.0	2.0	65536.0
20-47-65	Swine feces	non-ESBL	512.0	1.0	32.0	2.0	32768.0
20-47-66	Swine feces	non-ESBL	512.0	2.0	16.0	2.0	65536.0
20-47-67	Swine feces	non-ESBL	512.0	2.0	32.0	2.0	65536.0
20-47-7	Swine feces	ESBL	512.0	2.0	32.0	2.0	65536.0
20-47-8	Swine feces	ESBL	512.0	2.0	32.0	2.0	65536.0
20-47-83	Pork meat	non-ESBL	512.0	2.0	16.0	2.0	32768.0
20-47-84	Pork meat	non-ESBL	512.0	1.0	32.0	2.0	32768.0
20-47-85	Pork meat	non-ESBL	512.0	1.0	16.0	2.0	32768.0
20-47-86	Pork meat	ESBL	512.0	1.0	32.0	2.0	32768.0
20-47-87	Pork meat	ESBL	512.0	2.0	32.0	2.0	32768.0
20-47-88	Pork meat	ESBL	512.0	1.0	32.0	2.0	32768.0
20-47-89	Pork meat	non-ESBL	512.0	1.0	32.0	2.0	65536.0
20-47-9	Swine feces	ESBL	512.0	2.0	32.0	2.0	65536.0
20-47-90	Pork meat	non-ESBL	512.0	2.0	32.0	2.0	32768.0
20-47-91	Pork meat	non-ESBL	512.0	1.0	32.0	2.0	32768.0
20-47-92	Pork meat	ESBL	512.0	8.0	32.0	2.0	32768.0
20-47-93	Pork meat	non-ESBL	512.0	1.0	32.0	2.0	32768.0
20-47-94	Pork meat	ESBL	512.0	1.0	32.0	2.0	32768.0
20-47-95	Pork meat	ESBL	256.0	1.0	32.0	2.0	32768.0
20-47-96	Pork meat	non-ESBL	512.0	2.0	32.0	2.0	32768.0
20-47-97	Pork meat	non-ESBL	256.0	1.0	32.0	2.0	65536.0
20-47-98	Pork meat	non-ESBL	256.0	1.0	16.0	2.0	32768.0
20-47-99	Pork meat	non-ESBL	512.0	1.0	32.0	2.0	65536.0
21-47-10	Inpatient	ESBL	512.0	1.0	16.0	2.0	32768.0
21-47-100	Inpatient	ESBL	512.0	8.0	32.0	2.0	65536.0
21-47-101	Inpatient	ESBL	512.0	1.0	16.0	2.0	65536.0
21-47-102	Inpatient	non-ESBL	512.0	2.0	32.0	2.0	65536.0
21-47-103	Inpatient	ESBL	512.0	1.0	32.0	2.0	65536.0
21-47-104	Inpatient	ESBL	512.0	2.0	32.0	2.0	131072.0

21-47-105	Inpatient	non-ESBL	512.0	2.0	32.0	2.0	65536.0
21-47-106	Inpatient	non-ESBL	512.0	1.0	32.0	2.0	32768.0
21-47-107	Inpatient	non-ESBL	512.0	2.0	32.0	2.0	65536.0
21-47-108	Inpatient	non-ESBL	512.0	2.0	32.0	2.0	65536.0
21-47-109	Inpatient	non-ESBL	512.0	2.0	32.0	2.0	65536.0
21-47-11	Inpatient	ESBL	512.0	2.0	32.0	2.0	65536.0
21-47-110	Inpatient	non-ESBL	512.0	1.0	16.0	2.0	32768.0
21-47-111	Inpatient	non-ESBL	512.0	2.0	64.0	2.0	32768.0
21-47-112	Inpatient	non-ESBL	512.0	2.0	32.0	2.0	65536.0
21-47-113	Inpatient	non-ESBL	512.0	2.0	32.0	4.0	65536.0
21-47-114	Inpatient	non-ESBL	512.0	8.0	32.0	2.0	32768.0
21-47-115	Inpatient	non-ESBL	512.0	2.0	32.0	2.0	65536.0
21-47-116	Inpatient	non-ESBL	512.0	2.0	32.0	2.0	65536.0
21-47-117	Inpatient	non-ESBL	512.0	2.0	32.0	2.0	32768.0
21-47-118	Inpatient	non-ESBL	512.0	4.0	32.0	2.0	65536.0
21-47-119	Inpatient	non-ESBL	512.0	2.0	32.0	4.0	65536.0
21-47-12	Inpatient	ESBL	512.0	2.0	32.0	2.0	32768.0
21-47-121	Inpatient	non-ESBL	512.0	2.0	16.0	2.0	65536.0
21-47-122	Inpatient	non-ESBL	512.0	1.0	16.0	2.0	65536.0
21-47-123	Inpatient	non-ESBL	512.0	2.0	16.0	2.0	65536.0
21-47-124	Inpatient	non-ESBL	512.0	2.0	32.0	2.0	32768.0
21-47-125	Inpatient	non-ESBL	512.0	2.0	32.0	2.0	65536.0
21-47-126	Inpatient	non-ESBL	512.0	2.0	32.0	2.0	65536.0
21-47-127	Inpatient	non-ESBL	512.0	2.0	32.0	2.0	32768.0
21-47-128	Inpatient	non-ESBL	512.0	2.0	16.0	2.0	32768.0
21-47-129	Inpatient	non-ESBL	512.0	2.0	32.0	2.0	32768.0
21-47-13	Inpatient	ESBL	512.0	2.0	32.0	2.0	32768.0
21-47-14	Inpatient	ESBL	512.0	2.0	16.0	2.0	32768.0
21-47-15	Inpatient	ESBL	512.0	2.0	16.0	2.0	65536.0
21-47-16	Inpatient	ESBL	512.0	2.0	32.0	2.0	65536.0
21-47-17	Inpatient	ESBL	512.0	2.0	32.0	2.0	65536.0
21-47-19	Inpatient	ESBL	512.0	2.0	32.0	2.0	32768.0
21-47-2	Inpatient	ESBL	512.0	2.0	16.0	2.0	65536.0
21-47-20	Inpatient	ESBL	512.0	2.0	32.0	2.0	65536.0
21-47-22	Inpatient	ESBL	512.0	2.0	16.0	2.0	32768.0
21-47-23	Inpatient	ESBL	512.0	2.0	32.0	2.0	65536.0
21-47-25	Inpatient	ESBL	512.0	2.0	16.0	2.0	32768.0
21-47-26	Inpatient	ESBL	512.0	2.0	16.0	2.0	32768.0
21-47-27	Inpatient	ESBL	1024.0	2.0	32.0	2.0	65536.0
21-47-28	Inpatient	ESBL	512.0	2.0	32.0	2.0	65536.0
21-47-29	Inpatient	ESBL	512.0	2.0	16.0	1.0	32768.0
21-47-3	Inpatient	ESBL	512.0	2.0	16.0	2.0	65536.0
21-47-30	Inpatient	ESBL	512.0	2.0	16.0	2.0	65536.0
21-47-31	Inpatient	ESBL	512.0	2.0	32.0	2.0	32768.0
21-47-32	Inpatient	ESBL	512.0	2.0	32.0	2.0	32768.0
21-47-33	Inpatient	non-ESBL	512.0	2.0	8.0	2.0	16384.0
21-47-34	Inpatient	ESBL	256.0	2.0	32.0	2.0	65536.0
21-47-35	Inpatient	ESBL	512.0	2.0	16.0	2.0	65536.0
21-47-37	Inpatient	ESBL	512.0	2.0	32.0	2.0	65536.0
21-47-39	Inpatient	ESBL	512.0	2.0	32.0	2.0	32768.0
21-47-4	Inpatient	ESBL	512.0	2.0	16.0	2.0	65536.0

21-47-40	Inpatient	ESBL	512.0	2.0	16.0	2.0	65536.0
21-47-41	Inpatient	ESBL	512.0	2.0	16.0	2.0	65536.0
21-47-42	Inpatient	non-ESBL	512.0	2.0	16.0	2.0	65536.0
21-47-43	Inpatient	ESBL	512.0	2.0	16.0	2.0	65536.0
21-47-44	Inpatient	ESBL	512.0	2.0	16.0	2.0	65536.0
21-47-45	Inpatient	ESBL	1024.0	2.0	32.0	2.0	65536.0
21-47-46	Inpatient	ESBL	512.0	2.0	32.0	2.0	65536.0
21-47-47	Inpatient	ESBL	512.0	2.0	32.0	2.0	65536.0
21-47-48	Inpatient	ESBL	512.0	2.0	32.0	2.0	65536.0
21-47-49	Inpatient	ESBL	1024.0	2.0	32.0	2.0	65536.0
21-47-50	Inpatient	ESBL	512.0	2.0	32.0	2.0	65536.0
21-47-51	Inpatient	ESBL	512.0	2.0	16.0	2.0	65536.0
21-47-52	Inpatient	ESBL	512.0	2.0	32.0	2.0	65536.0
21-47-53	Inpatient	ESBL	512.0	2.0	32.0	2.0	32768.0
21-47-55	Inpatient	ESBL	256.0	2.0	32.0	2.0	32768.0
21-47-6	Inpatient	ESBL	512.0	2.0	16.0	2.0	32768.0
21-47-7	Inpatient	ESBL	512.0	2.0	16.0	2.0	32768.0
21-47-8	Inpatient	ESBL	512.0	2.0	16.0	2.0	32768.0
21-47-80	Inpatient	ESBL	256.0	2.0	64.0	2.0	32768.0
21-47-81	Inpatient	ESBL	512.0	2.0	64.0	2.0	32768.0
21-47-82	Inpatient	ESBL	512.0	2.0	64.0	2.0	65536.0
21-47-83	Inpatient	ESBL	512.0	4.0	128.0	2.0	65536.0
21-47-84	Inpatient	ESBL	512.0	2.0	64.0	2.0	65536.0
21-47-85	Inpatient	ESBL	512.0	4.0	64.0	2.0	32768.0
21-47-86	Inpatient	ESBL	512.0	2.0	64.0	2.0	32768.0
21-47-87	Inpatient	ESBL	512.0	2.0	32.0	2.0	65536.0
21-47-88	Inpatient	ESBL	512.0	2.0	64.0	2.0	32768.0
21-47-89	Inpatient	ESBL	512.0	1.0	32.0	2.0	65536.0
21-47-9	Inpatient	ESBL	512.0	2.0	16.0	2.0	65536.0
21-47-90	Inpatient	ESBL	512.0	2.0	32.0	2.0	65536.0
21-47-91	Inpatient	ESBL	512.0	2.0	64.0	4.0	32768.0
21-47-92	Inpatient	ESBL	512.0	1.0	64.0	2.0	65536.0
21-47-93	Inpatient	ESBL	1024.0	1.0	64.0	2.0	32768.0
21-47-94	Inpatient	ESBL	2048.0	1.0	64.0	2.0	65536.0
21-47-95	Inpatient	ESBL	512.0	2.0	64.0	2.0	32768.0
21-47-96	Inpatient	ESBL	512.0	2.0	16.0	2.0	65536.0
21-47-97	Inpatient	ESBL	1024.0	1.0	32.0	2.0	32768.0
21-47-98	Inpatient	ESBL	512.0	2.0	32.0	2.0	32768.0
21-47-99	Inpatient	ESBL	512.0	8.0	32.0	2.0	65536.0

MIC=minimum inhibitory concentration; MBC=minimum bactericidal concentration

GDA=glutaraldehyde; CHG=chlorhexidine digluconate; BAC=benzalkonium chloride; OCT=octenidine dihydrochloride; IPA=iso

ESBL=extended-spectrum beta-lactamase- producing *E. coli*

		MBC (mg/L)						
NaOCl	PCMC	GDA	CHG	BAC	OCT	IPA	NaOCl	PCMC
256.0	512.0	512.0	2.0	64.0	2.0	65536.0	512.0	1024.0
512.0	256.0	512.0	2.0	32.0	2.0	131072.0	1024.0	512.0
256.0	512.0	512.0	2.0	64.0	2.0	131072.0	512.0	1024.0
512.0	256.0	512.0	1.0	32.0	2.0	131072.0	1024.0	512.0
512.0	512.0	512.0	2.0	32.0	2.0	131072.0	512.0	1024.0
256.0	512.0	512.0	8.0	32.0	2.0	65536.0	512.0	1024.0
512.0	512.0	512.0	2.0	32.0	2.0	131072.0	512.0	1024.0
256.0	256.0	512.0	2.0	32.0	2.0	131072.0	512.0	512.0
512.0	256.0	512.0	8.0	32.0	2.0	131072.0	512.0	512.0
512.0	512.0	512.0	2.0	32.0	2.0	65536.0	512.0	512.0
256.0	256.0	512.0	2.0	16.0	2.0	131072.0	512.0	512.0
512.0	512.0	512.0	16.0	64.0	2.0	131072.0	512.0	512.0
256.0	256.0	512.0	2.0	32.0	2.0	131072.0	512.0	512.0
256.0	256.0	512.0	2.0	64.0	2.0	262144.0	256.0	512.0
256.0	256.0	512.0	4.0	64.0	2.0	65536.0	512.0	512.0
512.0	512.0	512.0	2.0	64.0	2.0	131072.0	512.0	512.0
512.0	256.0	512.0	4.0	32.0	2.0	131072.0	512.0	512.0
512.0	512.0	512.0	4.0	32.0	2.0	131072.0	512.0	512.0
256.0	256.0	512.0	2.0	32.0	2.0	131072.0	512.0	512.0
256.0	512.0	512.0	2.0	32.0	2.0	65536.0	512.0	512.0
512.0	512.0	512.0	2.0	32.0	2.0	131072.0	512.0	512.0
256.0	256.0	512.0	2.0	16.0	2.0	131072.0	512.0	512.0
512.0	512.0	512.0	2.0	32.0	2.0	131072.0	512.0	512.0
1024.0	256.0	512.0	1.0	32.0	2.0	131072.0	1024.0	512.0
256.0	256.0	512.0	2.0	16.0	4.0	65536.0	256.0	256.0
256.0	256.0	512.0	2.0	8.0	2.0	131072.0	512.0	512.0
512.0	256.0	256.0	1.0	32.0	2.0	65536.0	1024.0	512.0
1024.0	256.0	256.0	2.0	64.0	4.0	131072.0	1024.0	512.0
512.0	256.0	256.0	4.0	16.0	2.0	131072.0	512.0	512.0
512.0	256.0	256.0	2.0	32.0	2.0	65536.0	1024.0	512.0
256.0	256.0	512.0	2.0	32.0	2.0	131072.0	512.0	512.0
1024.0	256.0	512.0	2.0	32.0	2.0	131072.0	1024.0	512.0
256.0	256.0	512.0	2.0	64.0	4.0	65536.0	512.0	512.0
256.0	256.0	512.0	2.0	16.0	4.0	131072.0	256.0	512.0
1024.0	256.0	256.0	2.0	64.0	2.0	262144.0	1024.0	256.0
1024.0	256.0	512.0	2.0	32.0	2.0	131072.0	2048.0	512.0
256.0	256.0	512.0	2.0	16.0	8.0	131072.0	256.0	512.0
1024.0	256.0	256.0	2.0	32.0	2.0	131072.0	1024.0	512.0
512.0	256.0	512.0	2.0	16.0	2.0	65536.0	512.0	512.0
512.0	256.0	256.0	2.0	32.0	2.0	65536.0	1024.0	512.0
256.0	256.0	512.0	2.0	16.0	2.0	131072.0	512.0	512.0
512.0	256.0	512.0	2.0	16.0	2.0	65536.0	512.0	512.0
512.0	256.0	512.0	2.0	16.0	2.0	131072.0	512.0	1024.0
512.0	512.0	512.0	2.0	16.0	2.0	131072.0	1024.0	1024.0
512.0	256.0	512.0	2.0	16.0	2.0	131072.0	512.0	512.0
512.0	512.0	1024.0	4.0	16.0	4.0	131072.0	512.0	512.0
512.0	256.0	512.0	2.0	16.0	2.0	131072.0	512.0	512.0
512.0	512.0	512.0	2.0	16.0	2.0	131072.0	512.0	512.0

512.0	512.0	512.0	2.0	64.0	2.0	131072.0	512.0	512.0
256.0	256.0	1024.0	2.0	16.0	2.0	131072.0	512.0	512.0
512.0	512.0	512.0	2.0	32.0	2.0	131072.0	512.0	512.0
512.0	256.0	1024.0	2.0	16.0	4.0	131072.0	512.0	512.0
512.0	256.0	512.0	2.0	16.0	4.0	32768.0	512.0	512.0
512.0	256.0	1024.0	2.0	32.0	2.0	131072.0	512.0	512.0
512.0	512.0	1024.0	2.0	16.0	2.0	131072.0	512.0	512.0
512.0	512.0	2048.0	2.0	32.0	2.0	32768.0	512.0	512.0
256.0	256.0	1024.0	2.0	32.0	4.0	131072.0	512.0	512.0
512.0	256.0	512.0	2.0	16.0	2.0	262144.0	512.0	512.0
512.0	512.0	1024.0	2.0	16.0	2.0	131072.0	512.0	512.0
512.0	256.0	1024.0	2.0	32.0	2.0	32768.0	512.0	512.0
256.0	128.0	1024.0	1.0	32.0	1.0	16384.0	256.0	512.0
512.0	256.0	1024.0	2.0	32.0	2.0	131072.0	512.0	512.0
512.0	128.0	1024.0	2.0	32.0	4.0	65536.0	512.0	512.0
512.0	256.0	512.0	2.0	32.0	2.0	131072.0	512.0	512.0
256.0	256.0	1024.0	2.0	32.0	2.0	131072.0	512.0	512.0
512.0	256.0	1024.0	2.0	32.0	2.0	131072.0	512.0	512.0
512.0	256.0	512.0	2.0	32.0	2.0	131072.0	512.0	512.0
512.0	256.0	512.0	8.0	32.0	2.0	65536.0	1024.0	512.0
512.0	256.0	512.0	2.0	32.0	2.0	65536.0	512.0	512.0
512.0	256.0	1024.0	4.0	64.0	2.0	131072.0	512.0	512.0
256.0	256.0	512.0	4.0	32.0	8.0	65536.0	512.0	512.0
512.0	256.0	256.0	2.0	32.0	2.0	131072.0	1024.0	512.0
512.0	512.0	512.0	2.0	32.0	2.0	65536.0	1024.0	512.0
512.0	256.0	512.0	2.0	32.0	8.0	131072.0	512.0	512.0
512.0	256.0	512.0	16.0	64.0	2.0	131072.0	512.0	512.0
512.0	256.0	512.0	4.0	32.0	2.0	131072.0	512.0	512.0
512.0	256.0	1024.0	2.0	32.0	2.0	131072.0	512.0	512.0
512.0	256.0	512.0	2.0	32.0	2.0	131072.0	512.0	512.0
512.0	256.0	1024.0	2.0	32.0	4.0	131072.0	512.0	512.0
512.0	256.0	512.0	2.0	32.0	8.0	131072.0	512.0	512.0
512.0	512.0	1024.0	2.0	32.0	2.0	131072.0	512.0	512.0
512.0	256.0	512.0	2.0	32.0	2.0	131072.0	512.0	512.0
512.0	256.0	512.0	2.0	64.0	2.0	65536.0	512.0	512.0
512.0	256.0	512.0	2.0	32.0	2.0	131072.0	512.0	1024.0
256.0	256.0	512.0	2.0	32.0	2.0	131072.0	512.0	512.0
512.0	512.0	512.0	4.0	32.0	2.0	262144.0	512.0	512.0
512.0	256.0	512.0	2.0	32.0	2.0	131072.0	1024.0	512.0
512.0	512.0	1024.0	2.0	32.0	2.0	131072.0	512.0	512.0
512.0	512.0	512.0	2.0	32.0	2.0	131072.0	512.0	512.0
512.0	256.0	256.0	2.0	32.0	2.0	131072.0	512.0	1024.0
512.0	512.0	512.0	4.0	32.0	2.0	131072.0	1024.0	512.0
512.0	512.0	512.0	4.0	32.0	2.0	131072.0	512.0	1024.0
512.0	256.0	512.0	4.0	32.0	2.0	131072.0	512.0	512.0
512.0	256.0	512.0	2.0	32.0	2.0	131072.0	512.0	512.0
512.0	256.0	512.0	2.0	16.0	2.0	131072.0	512.0	512.0
512.0	256.0	512.0	2.0	32.0	2.0	65536.0	512.0	512.0
512.0	256.0	256.0	2.0	32.0	2.0	65536.0	1024.0	512.0
512.0	256.0	256.0	2.0	32.0	2.0	262144.0	1024.0	512.0
512.0	512.0	512.0	2.0	32.0	2.0	131072.0	512.0	512.0

512.0	256.0	512.0	1.0	64.0	2.0	131072.0	512.0	512.0
512.0	256.0	512.0	1.0	32.0	2.0	262144.0	512.0	512.0
256.0	256.0	512.0	2.0	32.0	2.0	262144.0	512.0	512.0
512.0	256.0	512.0	1.0	32.0	2.0	262144.0	512.0	512.0
1024.0	256.0	512.0	1.0	32.0	2.0	131072.0	1024.0	512.0
512.0	256.0	512.0	1.0	16.0	2.0	262144.0	512.0	512.0
512.0	256.0	512.0	1.0	64.0	2.0	262144.0	512.0	512.0
512.0	256.0	512.0	1.0	32.0	2.0	131072.0	512.0	512.0
512.0	256.0	512.0	1.0	32.0	2.0	262144.0	512.0	512.0
512.0	512.0	512.0	1.0	16.0	2.0	262144.0	512.0	512.0
512.0	512.0	512.0	1.0	32.0	2.0	131072.0	512.0	512.0
512.0	256.0	512.0	1.0	32.0	2.0	131072.0	512.0	512.0
512.0	256.0	512.0	1.0	64.0	8.0	262144.0	512.0	512.0
512.0	256.0	512.0	1.0	32.0	2.0	262144.0	1024.0	512.0
512.0	256.0	512.0	2.0	64.0	2.0	262144.0	512.0	512.0
512.0	512.0	512.0	1.0	32.0	2.0	262144.0	512.0	512.0
512.0	512.0	512.0	1.0	32.0	2.0	262144.0	512.0	512.0
512.0	256.0	512.0	1.0	64.0	4.0	262144.0	512.0	512.0
256.0	256.0	512.0	0.5	32.0	2.0	262144.0	512.0	512.0
512.0	256.0	512.0	1.0	32.0	4.0	131072.0	512.0	512.0
512.0	512.0	512.0	2.0	32.0	2.0	131072.0	512.0	512.0
512.0	256.0	512.0	2.0	32.0	2.0	32768.0	512.0	512.0
512.0	256.0	1024.0	4.0	32.0	4.0	65536.0	512.0	512.0
512.0	256.0	512.0	1.0	64.0	2.0	131072.0	512.0	512.0
512.0	256.0	256.0	1.0	32.0	2.0	65536.0	512.0	512.0
256.0	256.0	512.0	0.5	32.0	4.0	131072.0	512.0	512.0
512.0	256.0	256.0	1.0	32.0	2.0	131072.0	512.0	512.0
256.0	256.0	512.0	1.0	32.0	2.0	131072.0	512.0	512.0
512.0	512.0	512.0	0.5	32.0	2.0	131072.0	512.0	512.0
512.0	512.0	512.0	1.0	32.0	2.0	131072.0	512.0	512.0
512.0	512.0	512.0	0.5	32.0	2.0	131072.0	1024.0	512.0
512.0	512.0	512.0	2.0	64.0	4.0	262144.0	1024.0	512.0
256.0	256.0	512.0	0.5	32.0	2.0	131072.0	512.0	512.0
512.0	256.0	512.0	4.0	32.0	4.0	65536.0	512.0	512.0
512.0	512.0	512.0	0.5	128.0	2.0	131072.0	512.0	512.0
512.0	256.0	512.0	2.0	32.0	2.0	131072.0	512.0	512.0
256.0	256.0	512.0	1.0	32.0	2.0	131072.0	512.0	512.0
512.0	512.0	512.0	1.0	32.0	4.0	65536.0	512.0	512.0
512.0	256.0	512.0	1.0	32.0	2.0	131072.0	1024.0	512.0
512.0	256.0	512.0	1.0	32.0	8.0	262144.0	512.0	512.0
512.0	256.0	512.0	1.0	32.0	2.0	131072.0	512.0	512.0
512.0	256.0	512.0	1.0	32.0	2.0	131072.0	512.0	512.0
512.0	256.0	512.0	1.0	32.0	2.0	65536.0	512.0	512.0
512.0	256.0	512.0	1.0	64.0	2.0	131072.0	1024.0	512.0
512.0	256.0	512.0	1.0	16.0	2.0	131072.0	512.0	512.0
512.0	256.0	512.0	1.0	64.0	2.0	131072.0	512.0	512.0
512.0	256.0	512.0	1.0	128.0	2.0	262144.0	512.0	512.0
512.0	256.0	512.0	2.0	64.0	2.0	65536.0	512.0	512.0
512.0	512.0	512.0	1.0	64.0	4.0	131072.0	512.0	512.0
512.0	512.0	512.0	2.0	32.0	2.0	131072.0	512.0	512.0
512.0	256.0	512.0	1.0	32.0	8.0	65536.0	512.0	512.0

512.0	256.0	512.0	1.0	32.0	8.0	262144.0	512.0	512.0
512.0	512.0	512.0	1.0	64.0	2.0	131072.0	512.0	512.0
512.0	256.0	512.0	1.0	32.0	4.0	262144.0	512.0	512.0
512.0	256.0	512.0	2.0	32.0	2.0	131072.0	512.0	512.0
512.0	512.0	256.0	1.0	64.0	4.0	131072.0	512.0	512.0
512.0	256.0	512.0	1.0	32.0	4.0	131072.0	512.0	512.0
256.0	256.0	512.0	2.0	32.0	2.0	131072.0	512.0	512.0
256.0	256.0	512.0	4.0	16.0	2.0	131072.0	512.0	512.0
512.0	256.0	512.0	4.0	32.0	2.0	131072.0	1024.0	512.0
512.0	512.0	512.0	2.0	32.0	2.0	131072.0	512.0	512.0
512.0	256.0	512.0	4.0	32.0	2.0	131072.0	512.0	512.0
512.0	512.0	512.0	2.0	32.0	2.0	65536.0	512.0	512.0
512.0	256.0	512.0	2.0	32.0	2.0	131072.0	1024.0	512.0
256.0	256.0	512.0	2.0	64.0	2.0	131072.0	512.0	512.0
256.0	256.0	512.0	2.0	32.0	2.0	131072.0	512.0	512.0
256.0	256.0	512.0	2.0	32.0	2.0	131072.0	512.0	512.0
512.0	512.0	512.0	2.0	32.0	2.0	131072.0	512.0	512.0
256.0	256.0	1024.0	2.0	32.0	2.0	65536.0	512.0	512.0
512.0	256.0	512.0	2.0	32.0	2.0	65536.0	512.0	512.0
256.0	256.0	1024.0	2.0	32.0	2.0	131072.0	512.0	512.0
512.0	512.0	256.0	2.0	16.0	2.0	131072.0	512.0	512.0
512.0	512.0	512.0	2.0	64.0	2.0	65536.0	512.0	512.0
512.0	256.0	512.0	2.0	32.0	2.0	131072.0	512.0	512.0
256.0	256.0	512.0	2.0	64.0	8.0	131072.0	512.0	512.0
256.0	256.0	512.0	2.0	32.0	2.0	65536.0	512.0	512.0
512.0	256.0	512.0	2.0	64.0	2.0	131072.0	512.0	512.0
256.0	256.0	512.0	2.0	16.0	2.0	32768.0	512.0	512.0
512.0	256.0	512.0	2.0	16.0	2.0	131072.0	512.0	512.0
512.0	256.0	512.0	2.0	32.0	2.0	131072.0	512.0	512.0
256.0	256.0	512.0	2.0	32.0	2.0	131072.0	512.0	512.0
512.0	256.0	512.0	2.0	32.0	2.0	131072.0	512.0	512.0
512.0	256.0	512.0	2.0	32.0	2.0	131072.0	1024.0	512.0
512.0	512.0	512.0	2.0	32.0	2.0	131072.0	512.0	512.0
512.0	256.0	512.0	2.0	32.0	2.0	32768.0	512.0	512.0
512.0	256.0	512.0	2.0	32.0	2.0	131072.0	512.0	512.0
512.0	256.0	512.0	4.0	32.0	2.0	65536.0	512.0	512.0
512.0	256.0	512.0	2.0	32.0	2.0	131072.0	1024.0	512.0
512.0	256.0	512.0	2.0	32.0	2.0	131072.0	512.0	512.0
512.0	256.0	512.0	2.0	32.0	2.0	131072.0	512.0	512.0
512.0	256.0	512.0	4.0	32.0	2.0	131072.0	512.0	512.0
512.0	256.0	512.0	2.0	32.0	2.0	65536.0	512.0	512.0
512.0	512.0	256.0	2.0	32.0	4.0	131072.0	512.0	512.0
256.0	256.0	512.0	2.0	32.0	4.0	131072.0	512.0	512.0
512.0	256.0	512.0	2.0	32.0	2.0	32768.0	512.0	512.0
1024.0	256.0	512.0	2.0	32.0	2.0	65536.0	1024.0	512.0
1024.0	256.0	256.0	2.0	32.0	1.0	131072.0	1024.0	512.0
1024.0	256.0	512.0	2.0	32.0	2.0	131072.0	1024.0	512.0
512.0	512.0	512.0	2.0	32.0	2.0	131072.0	512.0	512.0
256.0	256.0	512.0	4.0	16.0	2.0	65536.0	512.0	512.0
512.0	512.0	512.0	2.0	32.0	2.0	131072.0	512.0	1024.0
512.0	512.0	512.0	2.0	32.0	2.0	131072.0	512.0	512.0

512.0	512.0	512.0	2.0	32.0	2.0	131072.0	512.0	512.0
512.0	256.0	512.0	4.0	64.0	2.0	131072.0	512.0	512.0
512.0	256.0	1024.0	2.0	64.0	2.0	131072.0	512.0	512.0
512.0	512.0	512.0	2.0	32.0	2.0	131072.0	512.0	512.0
1024.0	512.0	256.0	2.0	32.0	2.0	65536.0	1024.0	512.0
512.0	1024.0	512.0	2.0	64.0	4.0	131072.0	512.0	1024.0
512.0	256.0	512.0	2.0	32.0	2.0	65536.0	512.0	512.0
512.0	256.0	512.0	2.0	32.0	2.0	65536.0	1024.0	512.0
256.0	256.0	512.0	2.0	32.0	8.0	131072.0	512.0	512.0
512.0	512.0	512.0	2.0	32.0	2.0	131072.0	512.0	512.0
512.0	256.0	512.0	16.0	32.0	2.0	262144.0	1024.0	512.0
512.0	512.0	512.0	2.0	32.0	2.0	131072.0	512.0	512.0
512.0	256.0	512.0	2.0	64.0	2.0	131072.0	512.0	512.0
512.0	512.0	512.0	4.0	32.0	4.0	65536.0	512.0	512.0
512.0	512.0	512.0	2.0	16.0	2.0	131072.0	512.0	512.0
512.0	512.0	1024.0	2.0	32.0	2.0	131072.0	512.0	512.0
512.0	512.0	512.0	2.0	32.0	8.0	131072.0	512.0	512.0
512.0	256.0	512.0	2.0	64.0	2.0	65536.0	512.0	512.0
256.0	512.0	1024.0	4.0	64.0	8.0	131072.0	512.0	512.0
512.0	256.0	512.0	4.0	32.0	4.0	65536.0	512.0	512.0
512.0	256.0	512.0	2.0	64.0	2.0	65536.0	512.0	512.0
512.0	256.0	512.0	2.0	32.0	2.0	65536.0	512.0	512.0
512.0	256.0	512.0	4.0	64.0	2.0	262144.0	512.0	256.0
512.0	256.0	512.0	2.0	32.0	2.0	131072.0	512.0	512.0
256.0	512.0	512.0	2.0	64.0	2.0	131072.0	512.0	512.0
512.0	512.0	512.0	2.0	32.0	2.0	131072.0	512.0	1024.0
1024.0	256.0	512.0	1.0	32.0	2.0	131072.0	1024.0	512.0
512.0	512.0	512.0	2.0	32.0	2.0	131072.0	512.0	512.0
512.0	512.0	512.0	4.0	32.0	2.0	32768.0	512.0	1024.0
512.0	512.0	256.0	2.0	32.0	2.0	131072.0	512.0	512.0
512.0	512.0	512.0	2.0	32.0	2.0	131072.0	512.0	512.0
512.0	512.0	512.0	2.0	64.0	2.0	131072.0	512.0	512.0
256.0	512.0	512.0	2.0	16.0	2.0	131072.0	512.0	1024.0
256.0	256.0	512.0	4.0	32.0	2.0	65536.0	512.0	512.0
256.0	512.0	512.0	2.0	32.0	2.0	131072.0	512.0	1024.0
512.0	512.0	512.0	2.0	64.0	2.0	131072.0	512.0	1024.0
512.0	512.0	512.0	2.0	32.0	4.0	131072.0	512.0	1024.0
512.0	512.0	512.0	2.0	32.0	2.0	65536.0	512.0	512.0
512.0	512.0	512.0	2.0	32.0	2.0	131072.0	512.0	512.0
256.0	256.0	256.0	4.0	32.0	2.0	131072.0	512.0	512.0
256.0	256.0	512.0	2.0	32.0	2.0	131072.0	512.0	512.0
512.0	256.0	512.0	2.0	16.0	1.0	131072.0	512.0	512.0
512.0	256.0	256.0	2.0	16.0	2.0	131072.0	512.0	512.0
512.0	256.0	512.0	2.0	16.0	2.0	131072.0	512.0	512.0
512.0	256.0	512.0	2.0	16.0	2.0	65536.0	512.0	512.0
512.0	256.0	512.0	2.0	64.0	2.0	131072.0	512.0	512.0
512.0	256.0	512.0	8.0	32.0	2.0	131072.0	512.0	512.0
256.0	256.0	512.0	2.0	64.0	2.0	131072.0	512.0	512.0
256.0	256.0	512.0	2.0	16.0	1.0	65536.0	512.0	512.0
512.0	256.0	512.0	8.0	32.0	2.0	32768.0	512.0	512.0
512.0	512.0	512.0	2.0	32.0	4.0	65536.0	512.0	512.0

512.0	256.0	512.0	4.0	32.0	4.0	131072.0	512.0	512.0
512.0	256.0	512.0	2.0	32.0	2.0	65536.0	1024.0	1024.0
512.0	512.0	512.0	2.0	32.0	2.0	131072.0	512.0	512.0
512.0	256.0	512.0	4.0	32.0	2.0	131072.0	512.0	512.0
256.0	256.0	512.0	4.0	32.0	2.0	131072.0	512.0	512.0
1024.0	256.0	256.0	4.0	32.0	2.0		2048.0	512.0
256.0	256.0	512.0	2.0	16.0	2.0	32768.0	512.0	512.0
512.0	256.0	512.0	1.0	16.0	2.0	131072.0	512.0	512.0
512.0	256.0	1024.0	2.0	32.0	2.0	131072.0	512.0	512.0
512.0	256.0	512.0	2.0	32.0	2.0	131072.0	512.0	512.0
256.0	256.0	512.0	2.0	32.0	2.0	131072.0	512.0	512.0
512.0	512.0	512.0	2.0	32.0	2.0	65536.0	512.0	512.0
512.0	256.0	512.0	1.0	32.0	2.0	131072.0	1024.0	512.0
512.0	512.0	512.0	1.0	32.0	2.0	131072.0	512.0	512.0
512.0	512.0	512.0	1.0	32.0	2.0	131072.0	512.0	512.0
512.0	256.0	512.0	1.0	16.0	4.0	131072.0	512.0	512.0
512.0	256.0	512.0	2.0	32.0	2.0	131072.0	512.0	512.0
512.0	256.0	512.0	1.0	32.0	4.0	131072.0	512.0	512.0
512.0	256.0	256.0	2.0	32.0	2.0	131072.0	1024.0	512.0
512.0	256.0	256.0	1.0	16.0	2.0	131072.0	512.0	512.0
512.0	512.0	512.0	2.0	64.0	2.0	131072.0	512.0	512.0
512.0	512.0	512.0	2.0	32.0	2.0	131072.0	512.0	512.0
512.0	256.0	512.0	1.0	32.0	4.0	131072.0	512.0	512.0
512.0	256.0	512.0	2.0	16.0	2.0	131072.0	512.0	512.0
512.0	512.0	512.0	2.0	32.0	2.0	131072.0	512.0	512.0
512.0	256.0	512.0	2.0	32.0	2.0	131072.0	512.0	512.0
512.0	256.0	512.0	2.0	64.0	2.0	131072.0	1024.0	512.0
512.0	512.0	512.0	2.0	32.0	2.0	131072.0	512.0	512.0
512.0	512.0	512.0	1.0	32.0	2.0	131072.0	512.0	512.0
512.0	256.0	512.0	1.0	32.0	2.0	131072.0	512.0	512.0
256.0	256.0	512.0	1.0	32.0	2.0	131072.0	512.0	512.0
256.0	256.0	512.0	2.0	32.0	2.0	131072.0	512.0	512.0
512.0	256.0	512.0	1.0	32.0	2.0	131072.0	512.0	512.0
512.0	256.0	512.0	1.0	32.0	2.0	131072.0	512.0	512.0
512.0	512.0	512.0	2.0	32.0	2.0	131072.0	512.0	512.0
256.0	256.0	512.0	2.0	32.0	2.0	131072.0	512.0	512.0
512.0	256.0	512.0	1.0	32.0	2.0	131072.0	512.0	512.0
512.0	256.0	512.0	8.0	32.0	2.0	131072.0	512.0	512.0
256.0	256.0	512.0	2.0	32.0	4.0	131072.0	512.0	512.0
256.0	256.0	512.0	1.0	32.0	2.0	131072.0	512.0	512.0
512.0	256.0	256.0	1.0	32.0	2.0	32768.0	512.0	512.0
512.0	256.0	512.0	2.0	32.0	2.0	131072.0	512.0	512.0
512.0	256.0	256.0	1.0	32.0	2.0	131072.0	512.0	512.0
512.0	256.0	512.0	1.0	32.0	2.0	131072.0	512.0	512.0
512.0	256.0	512.0	1.0	32.0	2.0	131072.0	512.0	512.0
512.0	256.0	512.0	1.0	16.0	2.0	131072.0	512.0	512.0
512.0	512.0	512.0	8.0	32.0	2.0	131072.0	512.0	512.0
512.0	256.0	512.0	1.0	16.0	2.0	131072.0	512.0	512.0
512.0	256.0	512.0	2.0	32.0	2.0	131072.0	512.0	512.0
512.0	512.0	512.0	1.0	32.0	2.0	131072.0	512.0	512.0
512.0	256.0	1024.0	2.0	32.0	2.0	262144.0	512.0	512.0

512.0	256.0	1024.0	2.0	32.0	2.0	131072.0	512.0	512.0
512.0	256.0	512.0	2.0	32.0	2.0	65536.0	512.0	512.0
512.0	256.0	1024.0	2.0	32.0	2.0	131072.0	512.0	512.0
512.0	256.0	512.0	2.0	64.0	2.0	131072.0	512.0	512.0
512.0	256.0	512.0	2.0	32.0	2.0	65536.0	512.0	512.0
512.0	256.0	512.0	2.0	32.0	2.0	131072.0	512.0	512.0
512.0	256.0	512.0	1.0	16.0	2.0	131072.0	512.0	512.0
512.0	256.0	512.0	2.0	64.0	2.0	131072.0	512.0	512.0
512.0	256.0	512.0	2.0	32.0	2.0	131072.0	512.0	512.0
512.0	256.0	512.0	2.0	32.0	4.0	131072.0	512.0	512.0
512.0	256.0	512.0	8.0	32.0	4.0	131072.0	512.0	512.0
512.0	256.0	512.0	2.0	64.0	4.0	65536.0	512.0	512.0
512.0	512.0	512.0	2.0	32.0	2.0	65536.0	512.0	512.0
512.0	512.0	512.0	2.0	32.0	2.0	65536.0	512.0	512.0
512.0	512.0	512.0	32.0	32.0	2.0	131072.0	512.0	512.0
512.0	512.0	512.0	4.0	64.0	8.0	131072.0	512.0	512.0
512.0	512.0	512.0	2.0	32.0	4.0	131072.0	512.0	512.0
512.0	256.0	512.0	2.0	16.0	2.0	131072.0	512.0	512.0
512.0	256.0	512.0	1.0	64.0	2.0	131072.0	512.0	256.0
512.0	512.0	1024.0	2.0	32.0	8.0	131072.0	512.0	512.0
512.0	256.0	1024.0	2.0	32.0	2.0	131072.0	512.0	512.0
512.0	512.0	512.0	4.0	32.0	8.0	131072.0	512.0	512.0
512.0	256.0	512.0	4.0	32.0	2.0	131072.0	1024.0	512.0
512.0	512.0	512.0	4.0	32.0	2.0	32768.0	512.0	512.0
256.0	256.0	512.0	1.0	16.0	2.0	131072.0	512.0	512.0
512.0	256.0	512.0	4.0	32.0	2.0	131072.0	1024.0	256.0
512.0	256.0	512.0	2.0	32.0	2.0	65536.0	512.0	512.0
512.0	256.0	512.0	4.0	16.0	4.0	65536.0	512.0	512.0
512.0	256.0	512.0	2.0	16.0	2.0	131072.0	512.0	512.0
512.0	256.0	512.0	2.0	32.0	2.0	131072.0	512.0	512.0
512.0	512.0	512.0	2.0	32.0	2.0	131072.0	512.0	512.0
512.0	256.0	512.0	4.0	32.0	2.0	131072.0	512.0	512.0
512.0	256.0	512.0	2.0	16.0	2.0	65536.0	512.0	512.0
512.0	256.0	512.0	4.0	32.0	2.0	131072.0	512.0	512.0
512.0	256.0	512.0	2.0	16.0	2.0	131072.0	512.0	1024.0
512.0	512.0	512.0	2.0	32.0	2.0	131072.0	512.0	512.0
512.0	256.0	512.0	2.0	16.0	2.0	131072.0	512.0	512.0
512.0	256.0	1024.0	2.0	16.0	2.0	131072.0	512.0	512.0
512.0	256.0	1024.0	8.0	64.0	2.0	65536.0	512.0	512.0
512.0	512.0	512.0	2.0	64.0	2.0	131072.0	512.0	512.0
256.0	256.0	512.0	4.0	16.0	2.0	32768.0	512.0	512.0
512.0	256.0	512.0	2.0	32.0	2.0	131072.0	512.0	512.0
512.0	256.0	512.0	2.0	16.0	2.0	131072.0	512.0	512.0
512.0	256.0	512.0	8.0	32.0	2.0	131072.0	512.0	512.0
512.0	256.0	512.0	2.0	32.0	2.0	131072.0	512.0	512.0
256.0	128.0	512.0	2.0	8.0	4.0	32768.0	512.0	128.0
512.0	256.0	256.0	2.0	32.0	2.0	131072.0	512.0	512.0
512.0	256.0	512.0	2.0	16.0	2.0	131072.0	512.0	512.0
512.0	256.0	512.0	4.0	32.0	2.0	131072.0	512.0	512.0
512.0	256.0	512.0	4.0	32.0	2.0	131072.0	512.0	512.0
512.0	256.0	512.0	4.0	16.0	2.0	131072.0	512.0	512.0

512.0	256.0	512.0	2.0	16.0	2.0	65536.0	1024.0	512.0
512.0	256.0	512.0	2.0	16.0	2.0	131072.0	512.0	512.0
512.0	256.0	512.0	2.0	16.0	2.0	131072.0	1024.0	512.0
512.0	512.0	1024.0	4.0	32.0	2.0	131072.0	512.0	512.0
512.0	256.0	512.0	2.0	16.0	2.0	131072.0	512.0	512.0
512.0	256.0	1024.0	2.0	32.0	2.0	131072.0	512.0	512.0
512.0	256.0	512.0	2.0	32.0	2.0	131072.0	512.0	512.0
512.0	256.0	512.0	8.0	32.0	2.0	131072.0	512.0	512.0
512.0	256.0	512.0	4.0	32.0	2.0	131072.0	512.0	512.0
512.0	256.0	1024.0	4.0	32.0	2.0	131072.0	512.0	512.0
512.0	512.0	512.0	4.0	32.0	2.0	131072.0	1024.0	512.0
512.0	256.0	512.0	4.0	16.0	2.0	131072.0	512.0	512.0
512.0	256.0	512.0	4.0	32.0	2.0	131072.0	512.0	512.0
512.0	256.0	512.0	4.0	64.0	4.0	32768.0	512.0	512.0
512.0	256.0	256.0	2.0	32.0	2.0	131072.0	512.0	512.0
512.0	512.0	512.0	2.0	16.0	4.0	65536.0	512.0	512.0
512.0	256.0	512.0	2.0	32.0	2.0	65536.0	512.0	512.0
512.0	256.0	512.0	2.0	16.0	8.0	65536.0	512.0	512.0
512.0	256.0	256.0	2.0	64.0	2.0	131072.0	512.0	512.0
512.0	256.0	1024.0	4.0	64.0	2.0	131072.0	512.0	512.0
512.0	256.0	512.0	2.0	64.0	2.0	131072.0	512.0	512.0
512.0	512.0	512.0	4.0	128.0	4.0	131072.0	512.0	512.0
512.0	512.0	512.0	4.0	128.0	2.0	131072.0	512.0	512.0
512.0	512.0	1024.0	8.0	64.0	2.0	131072.0	512.0	512.0
512.0	256.0	512.0	2.0	64.0	2.0	131072.0	512.0	512.0
512.0	256.0	512.0	2.0	32.0	2.0	131072.0	512.0	512.0
256.0	512.0	512.0	2.0	64.0	2.0	65536.0	512.0	1024.0
512.0	512.0	512.0	1.0	32.0	8.0	65536.0	512.0	512.0
512.0	256.0	512.0	4.0	16.0	2.0	131072.0	512.0	1024.0
512.0	256.0	512.0	2.0	32.0	2.0	131072.0	512.0	512.0
256.0	256.0	512.0	2.0	64.0	8.0	32768.0	512.0	512.0
512.0	256.0	512.0	1.0	64.0	2.0	131072.0	512.0	512.0
512.0	256.0	1024.0	4.0	64.0	4.0	131072.0	512.0	512.0
512.0	512.0	2048.0	1.0	64.0	2.0	262144.0	512.0	512.0
512.0	256.0	512.0	2.0	64.0	2.0	131072.0	512.0	512.0
512.0	256.0	512.0	2.0	64.0	2.0	65536.0	1024.0	512.0
512.0	256.0	1024.0	2.0	32.0	2.0	131072.0	512.0	512.0
512.0	256.0	512.0	2.0	32.0	2.0	65536.0	512.0	512.0
512.0	512.0	512.0	8.0	32.0	2.0	131072.0	512.0	512.0

propanol; NaOCl=sodium hypochlorite; PCMC=chlorocresol.
