# Supplementary material for: Biocide Susceptibility and Antimicrobial Resistance of Escherichia coli Isolated from Swine Feces, Pork Meat and Humans in Germany
Source: Antibiotics (Basel). 2023 Apr 27;12(5):823. doi: 10.3390/antibiotics12050823 (PMC10215396; doi:10.3390/antibiotics12050823)
Supplement: Supplementary file 1 [file antibiotics-12-00823-s001.zip › antibiotics-2359558-supplementary/Table S2.pdf]

Table S2a. Pairwise comparisons of MIC distributions of *E. coli* of human and porcine origin.

Biocide	Origin of isolates	*p*-value			
		Swine feces	Pork meat	Voluntary donors	Inpatients
GDA	Swine feces				
	Pork meat	ns			
	Voluntary donors	ns	ns		
	Inpatients	**0.003**	**0.029**	ns	
CHG	Swine feces				
	Pork meat	**<0.0001**			
	Voluntary donors	ns	**<0.0001**		
	Inpatients	ns	**<0.0001**	ns	
BAC	Swine feces				
	Pork meat	ns			
	Voluntary donors	ns	ns		
	Inpatients	ns	ns	ns	
OCT	Swine feces				
	Pork meat	ns			
	Voluntary donors	ns	ns		
	Inpatients	ns	ns	ns	
IPA	Swine feces				
	Pork meat	ns			
	Voluntary donors	ns	ns		
	Inpatients	ns	ns	**0.039**	
NaOCl	Swine feces				
	Pork meat	ns			
	Voluntary donors	ns	ns		
	Inpatients	ns	**0.037**	ns	
PCMC	Swine feces				
	Pork meat	**0.001**			
	Voluntary donors	ns	ns		
	Inpatients	**0.007**	ns	ns	

GDA=glutaraldehyde; CHG=chlorhexidine digluconate; BAC=benzalkonium chloride; OCT=octenidine dihydrochloride; IPA=isoprop
Differences are considered significant at $p<0.05$. Significance values have been adjusted by the Bonferroni correction for multiple tests

panol; NaOCl=sodium hypochlorite; PCMC=chlorocresol; ns=not significant.

Table S2b. Pairwise comparisons of MBC distributions of *E. coli* of human and porcine origin

Biocide	Origin of isolates	***p*-value**			
		Swine feces	Pork meat	Voluntary donors	Inpatients
GDA	Swine feces				
	Pork meat	ns			
	Voluntary donors	ns	ns		
	Inpatients	**0.007**	**0.021**	ns	
CHG	Swine feces				
	Pork meat	**<0.0001**			
	Voluntary donors	ns	**<0.0001**		
	Inpatients	ns	**<0.0001**	ns	
BAC	Swine feces				
	Pork meat	ns			
	Voluntary donors	ns	ns		
	Inpatients	ns	ns	ns	
OCT	Swine feces				
	Pork meat	ns			
	Voluntary donors	ns	ns		
	Inpatients	ns	ns	ns	
IPA	Swine feces				
	Pork meat	**0.034**			
	Voluntary donors	ns	ns		
	Inpatients	ns	**0.019**	ns	
NaOCl	Swine feces				
	Pork meat	ns			
	Voluntary donors	ns	ns		
	Inpatients	ns	ns	ns	
PCMC	Swine feces				
	Pork meat	ns			
	Voluntary donors	ns	ns		
	Inpatients	ns	ns	ns	

GDA=glutaraldehyde; CHG=chlorhexidine digluconate; BAC=benzalkonium chloride; OCT=octenidine dihydrochloride; IPA=isopropanol;
Differences are considered significant at $p < 0.05$. Significance values have been adjusted by the Bonferroni correction for multiple tests.

; NaOCl=sodium hypochlorite; PCMC=chlorocresol; ns=not significant.
