# Supplementary material for: Biocide Susceptibility and Antimicrobial Resistance of Escherichia coli Isolated from Swine Feces, Pork Meat and Humans in Germany
Source: Antibiotics (Basel). 2023 Apr 27;12(5):823. doi: 10.3390/antibiotics12050823 (PMC10215396; doi:10.3390/antibiotics12050823)
Supplement: Supplementary file 1 [file antibiotics-12-00823-s001.zip › antibiotics-2359558-supplementary/Table S3.pdf]

Table S3a. Minimum inhibitory concentrations of biocides in non-ESBL and ESBL *E. coli* isolates.

Biocide	Resistance	0.25	0.5	1	2	4	8	16
GDA	ESBL							
	non-ESBL							
CHG	ESBL		3	41	153	13	4	2
	non-ESBL		4	40	122	10	1	-
BAC	ESBL				-	-	1	63
	non-ESBL				-	-	3	42
OCT	ESBL	-	-	5	209	2	-	-
	non-ESBL	-	-	2	169	6	-	-
IPA	ESBL							
	non-ESBL							
NaOCl	ESBL							
	non-ESBL							
PCMC	ESBL							
	non-ESBL							

GDA=glutaraldehyde; CHG=chlorhexidine digluconate; BAC=benzalkonium chloride; OCT=octenidine dihydrochloride; IPA=isoprop

MIC=minimum inhibitory concentration. MIC_{95}=The lowest concentration inhibiting growth of 95% of the bacterial population.

ESBL=extended-spectrum beta-lactamase-producing *E. coli*

Differences were considered significant at p <0.05.

Number of isolates with MIC values (mg/L) of								
**32**	**64**	**128**	**256**	**512**	**1024**	**2048**	**4096**	**8192**
	-	-	25	174	16	1	-	-
	-	-	27	144	6	-	-	-
-	-							
-	-							
135	16	1	-					
129	3	-	-					
-								
-								
						-	-	-
						-	-	-
	-	-	39	173	4	-	-	
	-	-	27	141	9	-	-	
	-	2	166	48	-	-	-	-
	-	1	113	62	1	-	-	-

panol; NaOCl=sodium hypochlorite; PCMC=chlorocresol.

16384	32768	65536	131072	262144	MIC_{95}	*p* -value
					1024	0.066
					512	
					4	0.176
					4	
					64	0.858
					32	
					2	0.066
					2	
7	115	90	4	-	65536	0.186
4	85	83	5	-	65536	
					512	0.186
					1024	
					512	0.003
					512	

Table S3b. Minimum bactericidal concentrations of biocides in non-ESBL and ESBL *E. coli* isolates.

Biocide	Resistance	0.25	0.5	1	2	4	8	16
GDA	ESBL							
	non-ESBL							
CHG	ESBL		2	34	134	32	12	2
	non-ESBL		4	39	107	24	1	1
BAC	ESBL				-	-	-	38
	non-ESBL				-	-	2	27
OCT	ESBL	-	-	3	184	22	7	-
	non-ESBL	-	-	1	144	21	11	-
IPA	ESBL							
	non-ESBL							
NaOCl	ESBL							
	non-ESBL							
PCMC	ESBL							
	non-ESBL							

GDA=glutaraldehyde; CHG=chlorhexidine digluconate; BAC=benzalkonium chloride; OCT=octenidine dihydrochloride; IPA=isop

MBC=minimum bactericidal concentration. MBC_{95}=lowest lethal concentration killing 95% of the bacterial population.

ESBL=extended-spectrum beta-lactamase-producing *E. coli*

Differences were considered significant at p <0.05.

Number of isolates with MBC values (mg/L) of								
**32**	**64**	**128**	**256**	**512**	**1024**	**2048**	**4096**	**8192**
	-	-	14	173	27	2	-	-
	-	-	15	150	12	-	-	-
-	-							
1	-							
139	37	2	-					
118	28	2	-					
-								
-								
						-	-	-
						-	-	-
	-	-	3	187	25	1	-	
	-	-	2	154	20	1	-	
	-	-	1	205	10	-	-	-
	-	1	4	160	12	-	-	-

ropanol; NaOCl=sodium hypochlorite; PCMC=chlorocresol.

16384	32768	65536	131072	262144	MBC_{95}	*p* -value
					1024	0.040
					1024	
					8	0.021
					4	
					64	0.988
					64	
					4	0.143
					8	
1	13	38	148	15	262144	0.153
-	3	30	131	13	262144	
					1024	0.984
					1024	
					512	0.965
					1024	
