# Supplementary material for: Biocide Susceptibility and Antimicrobial Resistance of Escherichia coli Isolated from Swine Feces, Pork Meat and Humans in Germany
Source: Antibiotics (Basel). 2023 Apr 27;12(5):823. doi: 10.3390/antibiotics12050823 (PMC10215396; doi:10.3390/antibiotics12050823)
Supplement: Supplementary file 1 [file antibiotics-12-00823-s001.zip › antibiotics-2359558-supplementary/Table S4.pdf]

Table S4. Antimicrobial susceptibility of 393 *E. coli*

**BfR-ID**	**Origin of isolates**	**Resistance**	AMP	AZI	CHL	CIP	COL	CTA
18-47-134	Voluntary donor	ESBL	>32	16	16	0.12	<=1	>4
18-47-135	Voluntary donor	ESBL	>32	8	64	>8	<=1	>4
18-47-136	Voluntary donor	ESBL	>32	4	16	8	<=1	>4
18-47-137	Voluntary donor	ESBL	>32	8	<=8	>8	<=1	>4
18-47-138	Voluntary donor	ESBL	>32	4	<=8	0.03	<=1	>4
18-47-139	Voluntary donor	ESBL	>32	4	<=8	8	<=1	>4
18-47-140	Voluntary donor	ESBL	>32	4	<=8	<=0.015	<=1	>4
18-47-141	Voluntary donor	ESBL	>32	8	<=8	<=0.015	<=1	>4
18-47-142	Voluntary donor	ESBL	>32	64	>64	8	<=1	>4
18-47-143	Voluntary donor	ESBL	>32	4	<=8	<=0.015	<=1	>4
18-47-144	Voluntary donor	ESBL	>32	8	<=8	<=0.015	<=1	>4
18-47-150	Voluntary donor	ESBL	>32	64	>64	8	<=1	>4
18-47-151	Voluntary donor	ESBL	>32	8	<=8	0.03	<=1	>4
18-47-152	Voluntary donor	ESBL	>32	4	<=8	<=0.015	<=1	>4
18-47-154	Voluntary donor	ESBL	>32	8	16	>8	<=1	>4
18-47-155	Voluntary donor	ESBL	>32	8	16	>8	<=1	>4
18-47-157	Voluntary donor	non-ESBL	4	8	<=8	<=0.015	<=1	<=0.25
18-47-158	Voluntary donor	non-ESBL	4	8	<=8	<=0.015	<=1	<=0.25
18-47-159	Voluntary donor	non-ESBL	>32	32	<=8	0.25	<=1	<=0.25
18-47-160	Voluntary donor	non-ESBL	2	8	<=8	<=0.015	<=1	<=0.25
18-47-161	Voluntary donor	non-ESBL	2	4	<=8	<=0.015	<=1	<=0.25
18-47-162	Voluntary donor	non-ESBL	4	4	<=8	<=0.015	<=1	<=0.25
18-47-164	Voluntary donor	non-ESBL	4	8	<=8	<=0.015	<=1	<=0.25
18-47-167	Voluntary donor	non-ESBL	4	8	<=8	<=0.015	<=1	<=0.25
18-47-168	Voluntary donor	non-ESBL	4	8	<=8	0.03	2	<=0.25
18-47-169	Voluntary donor	non-ESBL	2	4	<=8	<=0.015	<=1	<=0.25
18-47-170	Voluntary donor	non-ESBL	4	8	<=8	<=0.015	<=1	<=0.25
18-47-171	Voluntary donor	non-ESBL	>32	8	<=8	<=0.015	<=1	<=0.25
18-47-172	Voluntary donor	non-ESBL	2	8	<=8	<=0.015	<=1	<=0.25
18-47-174	Voluntary donor	ESBL	>32	4	<=8	>8	<=1	>4
18-47-176	Voluntary donor	ESBL	>32	8	<=8	<=0.015	<=1	>4
18-47-177	Voluntary donor	ESBL	>32	8	<=8	<=0.015	<=1	>4
18-47-178	Voluntary donor	ESBL	>32	8	<=8	>8	<=1	>4
18-47-179	Voluntary donor	ESBL	>32	64	<=8	<=0.015	<=1	>4
18-47-180	Voluntary donor	ESBL	>32	>64	>64	>8	<=1	>4
18-47-183	Voluntary donor	non-ESBL	4	8	<=8	0.03	<=1	<=0.25
18-47-184	Voluntary donor	non-ESBL	4	4	<=8	<=0.015	<=1	<=0.25
18-47-187	Voluntary donor	non-ESBL	>32	4	>64	0.25	<=1	<=0.25
18-47-188	Voluntary donor	non-ESBL	>32	4	>64	0.25	<=1	<=0.25
18-47-189	Voluntary donor	non-ESBL	4	4	<=8	<=0.015	<=1	<=0.25
18-47-190	Voluntary donor	non-ESBL	>32	4	<=8	<=0.015	<=1	<=0.25
18-47-191	Voluntary donor	non-ESBL	2	4	<=8	<=0.015	<=1	<=0.25
18-47-192	Voluntary donor	non-ESBL	2	4	<=8	<=0.015	<=1	<=0.25
18-47-193	Voluntary donor	non-ESBL	>32	4	>64	<=0.015	<=1	<=0.25
18-47-194	Voluntary donor	non-ESBL	>32	32	<=8	0.12	<=1	<=0.25
18-47-195	Voluntary donor	non-ESBL	4	8	<=8	<=0.015	<=1	<=0.25
18-47-196	Voluntary donor	non-ESBL	>32	4	<=8	0.25	4	<=0.25
18-47-197	Voluntary donor	non-ESBL	4	4	<=8	<=0.015	2	<=0.25

18-47-199	Voluntary donor	non-ESBL	4	16	<=8	<=0.015	2	<=0.25
18-47-202	Voluntary donor	non-ESBL	2	4	<=8	<=0.015	<=1	<=0.25
18-47-203	Voluntary donor	ESBL	>32	64	16	>8	<=1	>4
18-47-204	Voluntary donor	ESBL	>32	8	<=8	8	<=1	>4
18-47-208	Voluntary donor	ESBL	>32	64	<=8	>8	<=1	>4
18-47-209	Voluntary donor	ESBL	>32	4	<=8	4	<=1	>4
18-47-210	Voluntary donor	ESBL	>32	8	<=8	>8	<=1	>4
18-47-211	Voluntary donor	ESBL	>32	16	<=8	0.03	<=1	>4
18-47-213	Voluntary donor	ESBL	>32	8	<=8	<=0.015	<=1	>4
18-47-224	Voluntary donor	ESBL	>32	8	<=8	<=0.015	<=1	>4
18-47-225	Voluntary donor	non-ESBL	2	8	<=8	0.25	<=1	<=0.25
18-47-226	Voluntary donor	ESBL	>32	>64	<=8	>8	<=1	>4
18-47-227	Voluntary donor	ESBL	>32	>64	16	>8	<=1	>4
18-47-228	Voluntary donor	ESBL	>32	8	<=8	0.25	<=1	>4
18-47-230	Voluntary donor	ESBL	>32	>64	<=8	>8	<=1	>4
18-47-231	Voluntary donor	ESBL	>32	>64	<=8	>8	<=1	>4
18-47-233	Voluntary donor	ESBL	>32	8	<=8	0.25	<=1	>4
18-47-235	Voluntary donor	ESBL	>32	8	<=8	<=0.015	<=1	>4
18-47-239	Voluntary donor	ESBL	>32	>64	<=8	0.25	<=1	>4
18-47-241	Voluntary donor	ESBL	>32	>64	64	<=0.015	<=1	>4
18-47-242	Voluntary donor	ESBL	>32	64	<=8	>8	<=1	>4
18-47-244	Voluntary donor	ESBL	>32	8	>64	8	<=1	>4
18-47-246	Voluntary donor	ESBL	>32	64	<=8	0.03	<=1	>4
18-47-247	Voluntary donor	ESBL	>32	8	<=8	<=0.015	<=1	>4
18-47-248	Voluntary donor	ESBL	>32	8	>64	>8	<=1	>4
18-47-249	Voluntary donor	ESBL	>32	8	>64	8	<=1	>4
18-47-251	Voluntary donor	ESBL	>32	4	<=8	0.25	<=1	>4
18-47-252	Voluntary donor	ESBL	>32	8	<=8	<=0.015	<=1	>4
18-47-253	Voluntary donor	ESBL	>32	4	<=8	0.25	<=1	>4
18-47-342	Voluntary donor	non-ESBL	2	4	<=8	<=0.015	<=1	<=0.25
18-47-344	Voluntary donor	non-ESBL	8	8	16	0.03	<=1	<=0.25
18-47-345	Voluntary donor	non-ESBL	4	8	<=8	0.03	<=1	<=0.25
18-47-347	Voluntary donor	non-ESBL	>32	8	<=8	<=0.015	<=1	<=0.25
18-47-348	Voluntary donor	non-ESBL	2	4	<=8	0.25	<=1	<=0.25
18-47-349	Voluntary donor	non-ESBL	>32	64	<=8	<=0.015	<=1	<=0.25
18-47-350	Voluntary donor	non-ESBL	4	4	<=8	<=0.015	<=1	<=0.25
18-47-351	Voluntary donor	non-ESBL	2	4	<=8	<=0.015	<=1	<=0.25
18-47-352	Voluntary donor	non-ESBL	2	8	<=8	<=0.015	<=1	<=0.25
18-47-353	Voluntary donor	non-ESBL	4	8	<=8	0.03	<=1	<=0.25
18-47-358	Voluntary donor	non-ESBL	>32	4	<=8	<=0.015	<=1	<=0.25
18-47-359	Voluntary donor	non-ESBL	>32	4	<=8	<=0.015	<=1	<=0.25
18-47-360	Voluntary donor	non-ESBL	4	8	<=8	0.03	<=1	<=0.25
18-47-362	Voluntary donor	non-ESBL	4	8	<=8	0.03	4	<=0.25
18-47-363	Voluntary donor	non-ESBL	4	8	<=8	0.03	<=1	<=0.25
18-47-364	Voluntary donor	non-ESBL	4	8	<=8	<=0.015	<=1	<=0.25
18-47-369	Voluntary donor	ESBL	>32	>64	<=8	>8	<=1	>4
18-47-377	Voluntary donor	ESBL	>32	64	<=8	>8	<=1	>4
18-47-378	Voluntary donor	ESBL	>32	8	<=8	8	<=1	>4
18-47-229	Inpatient	ESBL	>32	16	16	>8	<=1	>4
18-47-232	Inpatient	ESBL	>32	4	<=8	<=0.015	<=1	>4
20-47-2	Swine feces	ESBL	>32	>64	>64	<=0.015	8	-

20-47-4	Swine feces	ESBL	>32	>64	>64	0.03	2	-
20-47-5	Swine feces	non-ESBL	2	8	<=8	<=0.015	<=1	<=0.25
20-47-6	Swine feces	non-ESBL	8	8	16	0.03	<=1	<=0.25
20-47-7	Swine feces	ESBL	>32	64	<=8	>8	<=1	>4
20-47-8	Swine feces	ESBL	>32	>64	<=8	>8	<=1	>4
20-47-9	Swine feces	ESBL	>32	8	16	1	<=1	>4
20-47-10	Swine feces	ESBL	>32	8	16	0.03	<=1	>4
20-47-11	Swine feces	ESBL	>32	8	16	0.03	<=1	>4
20-47-12	Swine feces	ESBL	>32	8	<=8	<=0.015	<=1	>4
20-47-13	Swine feces	ESBL	>32	8	16	<=0.015	<=1	>4
20-47-16	Swine feces	ESBL	>32	8	16	1	<=1	>4
20-47-18	Swine feces	ESBL	>32	8	<=8	<=0.015	<=1	>4
20-47-19	Swine feces	ESBL	>32	8	<=8	<=0.015	<=1	>4
20-47-20	Swine feces	ESBL	>32	8	<=8	<=0.015	<=1	>4
20-47-21	Swine feces	ESBL	>32	8	<=8	<=0.015	<=1	>4
20-47-23	Swine feces	ESBL	>32	8	<=8	0.5	<=1	>4
20-47-24	Swine feces	ESBL	>32	8	<=8	<=0.015	<=1	>4
20-47-25	Swine feces	ESBL	>32	16	16	1	<=1	>4
20-47-27	Swine feces	ESBL	>32	16	16	>8	<=1	>4
20-47-28	Swine feces	ESBL	>32	8	<=8	>8	<=1	>4
20-47-29	Swine feces	ESBL	>32	8	>64	8	8	>4
20-47-30	Swine feces	ESBL	>32	8	>64	8	4	>4
20-47-31	Swine feces	ESBL	>32	8	>64	>8	4	>4
20-47-32	Swine feces	ESBL	>32	32	>64	>8	8	>4
20-47-33	Swine feces	ESBL	>32	8	<=8	<=0.015	<=1	>4
20-47-34	Swine feces	ESBL	>32	8	<=8	<=0.015	2	>4
20-47-35	Swine feces	ESBL	>32	>64	>64	0.03	8	>4
20-47-36	Swine feces	ESBL	>32	>64	16	0.03	8	>4
20-47-37	Swine feces	ESBL	>32	>64	64	<=0.015	<=1	>4
20-47-38	Swine feces	ESBL	>32	>64	64	<=0.015	<=1	>4
20-47-40	Swine feces	ESBL	>32	>64	64	0.03	<=1	>4
20-47-41	Swine feces	ESBL	>32	4	<=8	<=0.015	<=1	>4
20-47-42	Swine feces	ESBL	>32	4	<=8	<=0.015	<=1	>4
20-47-43	Swine feces	ESBL	>32	4	<=8	<=0.015	<=1	>4
20-47-44	Swine feces	ESBL	>32	8	16	<=0.015	<=1	>4
20-47-45	Swine feces	ESBL	>32	4	<=8	<=0.015	<=1	>4
20-47-46	Swine feces	ESBL	>32	4	<=8	<=0.015	<=1	>4
20-47-47	Swine feces	ESBL	>32	4	64	<=0.015	<=1	>4
20-47-48	Swine feces	ESBL	>32	4	64	<=0.015	<=1	>4
20-47-49	Swine feces	ESBL	>32	4	<=8	<=0.015	<=1	>4
20-47-50	Swine feces	ESBL	>32	4	64	<=0.015	<=1	>4
20-47-51	Swine feces	ESBL	>32	8	<=8	0.03	<=1	>4
20-47-52	Swine feces	ESBL	>32	8	<=8	0.03	<=1	>4
20-47-53	Swine feces	ESBL	>32	4	<=8	<=0.015	2	>4
20-47-55	Swine feces	ESBL	>32	8	<=8	<=0.015	<=1	>4
20-47-56	Swine feces	ESBL	>32	8	<=8	<=0.015	<=1	>4
20-47-65	Swine feces	non-ESBL	>32	<=2	64	<=0.015	<=1	<=0.25
20-47-183	Swine feces	non-ESBL	2	8	<=8	0.25	<=1	<=0.25
20-47-184	Swine feces	non-ESBL	>64	>64	<=8	0.5	<=1	<=0.25
20-47-185	Swine feces	non-ESBL	>64	16	>128	<=0.015	<=1	<=0.25
20-47-186	Swine feces	non-ESBL	2	16	<=8	0.25	<=1	<=0.25

20-47-187	Swine feces	non-ESBL	4	16	<=8	<=0.015	<=1	<=0.25
20-47-188	Swine feces	non-ESBL	4	8	<=8	<=0.015	<=1	<=0.25
20-47-189	Swine feces	non-ESBL	<=1	8	<=8	<=0.015	<=1	<=0.25
20-47-190	Swine feces	non-ESBL	2	4	<=8	<=0.015	<=1	<=0.25
20-47-191	Swine feces	non-ESBL	4	8	<=8	<=0.015	<=1	<=0.25
20-47-192	Swine feces	non-ESBL	>64	8	<=8	0.03	<=1	<=0.25
20-47-193	Swine feces	non-ESBL	>64	16	<=8	<=0.015	<=1	<=0.25
20-47-194	Swine feces	non-ESBL	>64	8	<=8	<=0.015	<=1	<=0.25
20-47-195	Swine feces	non-ESBL	2	8	<=8	<=0.015	<=1	<=0.25
20-47-197	Swine feces	non-ESBL	2	8	<=8	<=0.015	<=1	<=0.25
20-47-198	Swine feces	non-ESBL	2	8	<=8	<=0.015	<=1	<=0.25
20-47-199	Swine feces	non-ESBL	>64	16	64	0.5	<=1	<=0.25
20-47-200	Swine feces	non-ESBL	>64	16	<=8	0.03	<=1	<=0.25
20-47-202	Swine feces	non-ESBL	4	16	<=8	0.03	<=1	<=0.25
20-47-203	Swine feces	non-ESBL	2	8	<=8	<=0.015	<=1	<=0.25
20-47-204	Swine feces	non-ESBL	4	8	<=8	<=0.015	<=1	<=0.25
20-47-205	Swine feces	non-ESBL	8	8	16	0.03	<=1	<=0.25
20-47-206	Swine feces	non-ESBL	2	8	<=8	0.03	<=1	<=0.25
20-47-207	Swine feces	non-ESBL	4	4	<=8	0.03	<=1	<=0.25
20-47-208	Swine feces	non-ESBL	<=1	8	<=8	<=0.015	<=1	<=0.25
20-47-209	Swine feces	non-ESBL	>64	4	<=8	<=0.015	<=1	<=0.25
20-47-210	Swine feces	non-ESBL	2	8	<=8	<=0.015	<=1	<=0.25
20-47-211	Swine feces	non-ESBL	>64	16	<=8	<=0.015	<=1	<=0.25
20-47-212	Swine feces	non-ESBL	8	8	<=8	0.03	<=1	<=0.25
20-47-213	Swine feces	non-ESBL	>64	8	128	0.03	4	<=0.25
20-47-214	Swine feces	non-ESBL	2	<=2	<=8	0.5	<=1	<=0.25
20-47-215	Swine feces	non-ESBL	4	8	16	0.03	<=1	<=0.25
20-47-216	Swine feces	non-ESBL	>64	4	<=8	<=0.015	<=1	<=0.25
20-47-217	Swine feces	non-ESBL	4	8	<=8	<=0.015	<=1	<=0.25
20-47-219	Swine feces	non-ESBL	>64	8	<=8	<=0.015	<=1	<=0.25
20-47-220	Swine feces	non-ESBL	4	8	<=8	<=0.015	<=1	<=0.25
20-47-222	Swine feces	non-ESBL	4	16	16	0.03	<=1	<=0.25
20-47-223	Swine feces	non-ESBL	2	4	<=8	>8	<=1	<=0.25
20-47-224	Swine feces	non-ESBL	2	4	<=8	<=0.015	<=1	<=0.25
20-47-225	Swine feces	non-ESBL	4	8	<=8	<=0.015	<=1	<=0.25
20-47-226	Swine feces	non-ESBL	4	8	<=8	>8	<=1	<=0.25
20-47-227	Swine feces	non-ESBL	>64	16	<=8	<=0.015	<=1	<=0.25
20-47-228	Swine feces	non-ESBL	4	8	<=8	0.03	<=1	<=0.25
20-47-229	Swine feces	non-ESBL	2	8	<=8	0.03	<=1	<=0.25
20-47-230	Swine feces	non-ESBL	>64	8	<=8	<=0.015	<=1	<=0.25
20-47-231	Swine feces	non-ESBL	2	<=2	<=8	<=0.015	<=1	<=0.25
20-47-232	Swine feces	non-ESBL	2	4	<=8	<=0.015	<=1	<=0.25
20-47-233	Swine feces	non-ESBL	2	8	<=8	<=0.015	<=1	<=0.25
20-47-66	Swine feces	non-ESBL	>32	4	64	<=0.015	<=1	<=0.25
20-47-58	Swine feces	ESBL	>32	8	<=8	<=0.015	<=1	>4
20-47-59	Swine feces	ESBL	>32	8	<=8	<=0.015	<=1	>4
20-47-60	Swine feces	ESBL	>32	4	<=8	<=0.015	<=1	>4
20-47-67	Swine feces	non-ESBL	>32	8	<=8	<=0.015	<=1	<=0.25
20-47-83	Pork meat	non-ESBL	2	8	<=8	<=0.015	<=1	<=0.25
20-47-84	Pork meat	non-ESBL	2	16	<=8	<=0.015	<=1	<=0.25
20-47-85	Pork meat	non-ESBL	2	8	<=8	<=0.015	<=1	<=0.25

20-47-86	Pork meat	ESBL	>64	64	>128	<=0.015	<=1	>4
20-47-87	Pork meat	ESBL	>64	16	16	8	<=1	>4
20-47-88	Pork meat	ESBL	>64	4	<=8	0.06	<=1	>4
20-47-89	Pork meat	non-ESBL	>64	16	<=8	<=0.015	<=1	<=0.25
20-47-90	Pork meat	non-ESBL	>64	16	32	0.03	<=1	<=0.25
20-47-91	Pork meat	ESBL	>64	4	<=8	<=0.015	<=1	1
20-47-92	Pork meat	ESBL	>64	32	<=8	0.5	2	>4
20-47-93	Pork meat	non-ESBL	>64	8	<=8	0.25	<=1	<=0.25
20-47-94	Pork meat	ESBL	>64	16	>128	1	<=1	>4
20-47-95	Pork meat	ESBL	>64	4	<=8	0.03	<=1	>4
20-47-96	Pork meat	non-ESBL	>64	16	64	0.03	<=1	<=0.25
20-47-97	Pork meat	non-ESBL	>64	16	<=8	<=0.015	<=1	<=0.25
20-47-98	Pork meat	non-ESBL	2	8	<=8	<=0.015	<=1	<=0.25
20-47-99	Pork meat	non-ESBL	4	8	<=8	0.03	<=1	<=0.25
20-47-100	Pork meat	non-ESBL	>64	16	<=8	0.03	<=1	<=0.25
20-47-101	Pork meat	non-ESBL	4	16	<=8	0.03	<=1	<=0.25
20-47-102	Pork meat	ESBL	>64	4	<=8	<=0.015	<=1	>4
20-47-103	Pork meat	ESBL	>64	4	<=8	<=0.015	<=1	>4
20-47-104	Pork meat	ESBL	>64	8	<=8	<=0.015	<=1	>4
20-47-105	Pork meat	non-ESBL	2	4	<=8	<=0.015	<=1	<=0.25
20-47-106	Pork meat	non-ESBL	4	4	<=8	<=0.015	<=1	<=0.25
20-47-107	Pork meat	ESBL	>64	32	>128	0.25	<=1	>4
20-47-108	Pork meat	ESBL	>64	16	<=8	<=0.015	<=1	>4
20-47-109	Pork meat	non-ESBL	4	8	<=8	<=0.015	<=1	<=0.25
20-47-110	Pork meat	ESBL	>64	32	<=8	<=0.015	<=1	>4
20-47-111	Pork meat	ESBL	>64	>64	16	<=0.015	<=1	1
20-47-112	Pork meat	ESBL	>64	8	>128	<=0.015	<=1	>4
20-47-113	Pork meat	ESBL	>64	16	<=8	0.5	<=1	>4
20-47-114	Pork meat	ESBL	>64	64	128	>8	<=1	>4
20-47-115	Pork meat	non-ESBL	4	16	<=8	0.06	<=1	<=0.25
20-47-116	Pork meat	non-ESBL	2	16	16	<=0.015	<=1	<=0.25
20-47-117	Pork meat	non-ESBL	>64	8	64	0.03	<=1	<=0.25
20-47-119	Pork meat	non-ESBL	4	4	<=8	<=0.015	<=1	<=0.25
20-47-120	Pork meat	ESBL	>64	<=2	<=8	<=0.015	<=1	>4
20-47-122	Pork meat	ESBL	>64	4	<=8	>8	<=1	>4
20-47-123	Pork meat	non-ESBL	4	8	16	<=0.015	<=1	<=0.25
20-47-124	Pork meat	ESBL	>64	4	<=8	<=0.015	<=1	>4
20-47-125	Pork meat	non-ESBL	8	4	16	<=0.015	<=1	<=0.25
20-47-126	Pork meat	non-ESBL	8	4	<=8	<=0.015	<=1	<=0.25
20-47-127	Pork meat	non-ESBL	2	8	<=8	<=0.015	<=1	<=0.25
20-47-128	Pork meat	non-ESBL	>64	8	<=8	0.25	<=1	<=0.25
20-47-129	Pork meat	non-ESBL	4	8	<=8	<=0.015	<=1	<=0.25
20-47-130	Pork meat	ESBL	>64	4	<=8	<=0.015	<=1	>4
20-47-131	Pork meat	non-ESBL	4	8	<=8	<=0.015	<=1	<=0.25
20-47-132	Pork meat	non-ESBL	>64	8	<=8	<=0.015	<=1	<=0.25
20-47-133	Pork meat	non-ESBL	>64	8	>128	<=0.015	<=1	<=0.25
20-47-134	Pork meat	non-ESBL	4	8	<=8	0.03	<=1	<=0.25
20-47-135	Pork meat	non-ESBL	>64	8	32	<=0.015	<=1	<=0.25
20-47-136	Pork meat	ESBL	>64	8	<=8	<=0.015	<=1	>4
20-47-137	Pork meat	non-ESBL	4	8	<=8	<=0.015	<=1	<=0.25
20-47-138	Pork meat	non-ESBL	4	8	<=8	0.03	<=1	<=0.25

20-47-139	Pork meat	ESBL	>64	8	<=8	0.25	<=1	>4
20-47-140	Pork meat	ESBL	>64	8	<=8	<=0.015	<=1	1
20-47-141	Pork meat	non-ESBL	4	8	<=8	<=0.015	<=1	<=0.25
20-47-142	Pork meat	non-ESBL	4	4	<=8	<=0.015	<=1	<=0.25
20-47-143	Pork meat	non-ESBL	4	8	<=8	<=0.015	<=1	<=0.25
20-47-144	Pork meat	non-ESBL	4	4	<=8	<=0.015	<=1	<=0.25
20-47-145	Pork meat	non-ESBL	>64	8	>128	<=0.015	<=1	<=0.25
20-47-146	Pork meat	non-ESBL	4	4	<=8	<=0.015	<=1	<=0.25
20-47-147	Pork meat	non-ESBL	4	4	<=8	<=0.015	<=1	<=0.25
20-47-148	Pork meat	non-ESBL	4	8	<=8	<=0.015	<=1	<=0.25
20-47-149	Pork meat	non-ESBL	>64	4	<=8	<=0.015	<=1	<=0.25
20-47-150	Pork meat	non-ESBL	2	4	<=8	<=0.015	<=1	<=0.25
20-47-151	Pork meat	ESBL	>64	4	<=8	<=0.015	<=1	>4
20-47-152	Pork meat	non-ESBL	>64	8	>128	<=0.015	<=1	<=0.25
20-47-153	Pork meat	non-ESBL	>64	8	<=8	<=0.015	<=1	<=0.25
20-47-154	Pork meat	non-ESBL	4	4	<=8	<=0.015	<=1	<=0.25
20-47-155	Pork meat	non-ESBL	4	4	<=8	<=0.015	<=1	<=0.25
20-47-156	Pork meat	non-ESBL	<=1	8	<=8	0.25	<=1	<=0.25
20-47-157	Pork meat	ESBL	>64	8	<=8	>8	<=1	>4
20-47-158	Pork meat	non-ESBL	4	16	16	0.03	<=1	<=0.25
20-47-159	Pork meat	non-ESBL	4	16	<=8	<=0.015	<=1	<=0.25
20-47-160	Pork meat	non-ESBL	4	8	<=8	<=0.015	<=1	<=0.25
20-47-161	Pork meat	ESBL	>64	8	<=8	<=0.015	<=1	>4
20-47-162	Pork meat	ESBL	>64	4	<=8	<=0.015	<=1	>4
20-47-163	Pork meat	non-ESBL	2	8	<=8	<=0.015	<=1	<=0.25
20-47-164	Pork meat	ESBL	>64	8	<=8	<=0.015	<=1	>4
20-47-165	Pork meat	ESBL	>64	8	<=8	<=0.015	<=1	>4
20-47-166	Pork meat	ESBL	>64	8	16	>8	<=1	>4
20-47-167	Pork meat	non-ESBL	>64	8	64	<=0.015	<=1	<=0.25
20-47-168	Pork meat	non-ESBL	>64	4	<=8	<=0.015	4	<=0.25
20-47-169	Pork meat	ESBL	>64	>64	<=8	<=0.015	<=1	>4
20-47-170	Pork meat	ESBL	>64	16	<=8	<=0.015	<=1	>4
20-47-171	Pork meat	ESBL	>64	4	<=8	0.25	<=1	>4
20-47-173	Pork meat	ESBL	>64	8	<=8	<=0.015	<=1	>4
20-47-174	Pork meat	ESBL	>64	32	<=8	8	<=1	>4
20-47-175	Pork meat	ESBL	>64	4	<=8	0.03	<=1	>4
20-47-176	Pork meat	ESBL	>64	8	<=8	<=0.015	<=1	>4
20-47-177	Pork meat	ESBL	>64	8	<=8	<=0.015	<=1	>4
20-47-178	Pork meat	ESBL	>64	64	<=8	>8	<=1	>4
20-47-179	Pork meat	ESBL	>64	8	<=8	<=0.015	<=1	>4
20-47-180	Pork meat	ESBL	>64	4	<=8	<=0.015	<=1	>4
20-47-181	Pork meat	non-ESBL	2	4	<=8	<=0.015	<=1	<=0.25
20-47-182	Pork meat	non-ESBL	4	8	32	<=0.015	<=1	<=0.25
20-47-121	Pork meat	ESBL	>64	8	<=8	<=0.015	<=1	>4
20-47-172	Pork meat	ESBL	>64	8	<=8	<=0.015	<=1	2
21-47-2	Inpatient	ESBL	>32	4	<=8	<=0.015	<=1	>4
21-47-3	Inpatient	ESBL	>32	32	<=8	1	<=1	>4
21-47-4	Inpatient	ESBL	>32	>64	<=8	<=0.015	<=1	>4
21-47-6	Inpatient	ESBL	>32	4	<=8	<=0.015	<=1	>4
21-47-7	Inpatient	ESBL	>32	8	<=8	<=0.015	<=1	>4
21-47-8	Inpatient	ESBL	>32	4	<=8	<=0.015	<=1	>4

21-47-9	Inpatient	ESBL	>32	4	<=8	4	<=1	>4
21-47-10	Inpatient	ESBL	>32	<=2	>64	<=0.015	<=1	>4
21-47-11	Inpatient	ESBL	>32	8	>64	>8	<=1	>4
21-47-12	Inpatient	ESBL	>32	8	<=8	<=0.015	<=1	>4
21-47-13	Inpatient	ESBL	>32	32	<=8	>8	<=1	>4
21-47-14	Inpatient	ESBL	>32	>64	<=8	0.12	<=1	>4
21-47-15	Inpatient	ESBL	>32	8	<=8	<=0.015	<=1	>4
21-47-16	Inpatient	ESBL	>32	4	<=8	0.25	<=1	>4
21-47-17	Inpatient	ESBL	>32	64	>64	>8	<=1	>4
21-47-19	Inpatient	ESBL	>32	4	16	>8	<=1	>4
21-47-20	Inpatient	ESBL	>32	32	<=8	>8	<=1	>4
21-47-22	Inpatient	ESBL	>32	32	<=8	>8	2	>4
21-47-23	Inpatient	ESBL	>32	>64	16	0.25	<=1	>4
21-47-25	Inpatient	ESBL	>32	4	<=8	0.25	<=1	>4
21-47-26	Inpatient	ESBL	>32	32	<=8	>8	<=1	>4
21-47-27	Inpatient	ESBL	>32	32	<=8	>8	<=1	>4
21-47-28	Inpatient	ESBL	>32	16	32	1	<=1	>4
21-47-29	Inpatient	ESBL	>32	4	>64	>8	<=1	>4
21-47-30	Inpatient	ESBL	>32	8	16	>8	<=1	>4
21-47-31	Inpatient	ESBL	>32	32	64	>8	<=1	>4
21-47-32	Inpatient	ESBL	>32	32	<=8	>8	<=1	>4
21-47-33	Inpatient	non-ESBL	4	32	<=8	>8	<=1	<=0.25
21-47-34	Inpatient	ESBL	>32	8	<=8	>8	<=1	>4
21-47-35	Inpatient	ESBL	>32	64	<=8	>8	<=1	>4
21-47-37	Inpatient	ESBL	>32	>64	<=8	0.5	<=1	>4
21-47-39	Inpatient	ESBL	>32	8	<=8	>8	<=1	>4
21-47-40	Inpatient	ESBL	>32	<=2	<=8	4	<=1	>4
21-47-41	Inpatient	ESBL	>32	8	<=8	>8	<=1	>4
21-47-42	Inpatient	ESBL	>32	4	>64	>8	<=1	1
21-47-43	Inpatient	ESBL	>32	8	>64	>8	<=1	>4
21-47-44	Inpatient	ESBL	>32	4	<=8	>8	<=1	>4
21-47-45	Inpatient	ESBL	>32	8	<=8	>8	<=1	>4
21-47-46	Inpatient	ESBL	>32	4	<=8	>8	<=1	>4
21-47-47	Inpatient	ESBL	>32	4	<=8	>8	<=1	>4
21-47-48	Inpatient	ESBL	>32	4	<=8	>8	<=1	>4
21-47-49	Inpatient	ESBL	>32	32	<=8	1	<=1	>4
21-47-50	Inpatient	ESBL	>32	16	32	4	<=1	>4
21-47-51	Inpatient	ESBL	>32	>64	>64	8	<=1	4
21-47-52	Inpatient	ESBL	>32	32	<=8	>8	<=1	>4
21-47-53	Inpatient	ESBL	>32	64	<=8	>8	<=1	>4
21-47-55	Inpatient	ESBL	>32	16	<=8	>8	<=1	>4
21-47-80	Inpatient	ESBL	>32	64	16	>8	<=1	>4
21-47-81	Inpatient	ESBL	>32	>64	<=8	>8	<=1	>4
21-47-82	Inpatient	ESBL	>32	64	16	>8	<=1	>4
21-47-83	Inpatient	ESBL	>32	16	32	0.5	<=1	>4
21-47-84	Inpatient	ESBL	>32	16	16	0.06	<=1	>4
21-47-85	Inpatient	ESBL	>32	8	64	>8	<=1	>4
21-47-86	Inpatient	ESBL	>32	4	<=8	>8	<=1	>4
21-47-87	Inpatient	ESBL	>32	64	<=8	>8	<=1	>4
21-47-88	Inpatient	ESBL	>32	8	16	>8	<=1	>4
21-47-89	Inpatient	ESBL	>32	4	>64	>8	<=1	>4

21-47-90	Inpatient	ESBL	>32	8	<=8	8	<=1	>4
21-47-91	Inpatient	ESBL	>32	4	<=8	>8	<=1	>4
21-47-92	Inpatient	ESBL	>32	64	<=8	0.06	<=1	>4
21-47-93	Inpatient	ESBL	>32	4	<=8	1	2	>4
21-47-94	Inpatient	ESBL	>32	8	<=8	<=0.015	<=1	>4
21-47-95	Inpatient	ESBL	>32	8	<=8	4	<=1	>4
21-47-96	Inpatient	ESBL	>32	8	16	>8	<=1	>4
21-47-97	Inpatient	ESBL	>32	64	<=8	>8	<=1	>4
21-47-98	Inpatient	ESBL	>32	4	<=8	>8	<=1	>4
21-47-99	Inpatient	ESBL	>32	8	>64	8	<=1	>4
21-47-100	Inpatient	ESBL	>32	8	>64	8	<=1	>4
21-47-101	Inpatient	ESBL	>32	4	64	<=0.015	<=1	>4
21-47-102	Inpatient	non-ESBL	4	8	<=8	>8	<=1	<=0.25
21-47-103	Inpatient	ESBL	>32	8	<=8	2	<=1	>4
21-47-104	Inpatient	ESBL	>32	4	<=8	<=0.015	<=1	>4
21-47-105	Inpatient	non-ESBL	>32	8	<=8	0.03	<=1	<=0.25
21-47-106	Inpatient	non-ESBL	>32	8	16	>8	<=1	<=0.25
21-47-107	Inpatient	non-ESBL	>32	8	<=8	<=0.015	<=1	<=0.25
21-47-108	Inpatient	non-ESBL	>32	16	<=8	>8	<=1	<=0.25
21-47-109	Inpatient	non-ESBL	2	4	<=8	<=0.015	<=1	<=0.25
21-47-110	Inpatient	non-ESBL	>32	4	<=8	0.25	<=1	<=0.25
21-47-111	Inpatient	non-ESBL	>32	8	16	>8	<=1	<=0.25
21-47-112	Inpatient	non-ESBL	>32	64	>64	0.5	<=1	<=0.25
21-47-113	Inpatient	non-ESBL	>32	8	>64	>8	<=1	<=0.25
21-47-114	Inpatient	non-ESBL	>32	64	>64	>8	<=1	<=0.25
21-47-115	Inpatient	non-ESBL	>32	4	<=8	0.25	<=1	<=0.25
21-47-116	Inpatient	non-ESBL	>32	32	<=8	>8	<=1	<=0.25
21-47-117	Inpatient	non-ESBL	4	4	<=8	0.03	<=1	<=0.25
21-47-118	Inpatient	non-ESBL	>32	4	<=8	8	<=1	<=0.25
21-47-119	Inpatient	non-ESBL	>32	8	<=8	<=0.015	<=1	<=0.25
21-47-121	Inpatient	non-ESBL	2	4	<=8	0.03	<=1	<=0.25
21-47-122	Inpatient	non-ESBL	>32	4	<=8	0.03	<=1	<=0.25
21-47-123	Inpatient	non-ESBL	2	4	<=8	0.03	<=1	<=0.25
21-47-124	Inpatient	non-ESBL	>32	4	<=8	<=0.015	<=1	<=0.25
21-47-125	Inpatient	non-ESBL	4	64	>64	>8	<=1	<=0.25
21-47-126	Inpatient	ESBL	>32	8	16	>8	<=1	>4
21-47-127	Inpatient	non-ESBL	>32	4	<=8	0.03	<=1	<=0.25
21-47-128	Inpatient	non-ESBL	4	8	<=8	<=0.015	<=1	<=0.25
21-47-129	Inpatient	non-ESBL	4	4	<=8	0.25	2	<=0.25

AMP=ampicillin; AZI=azithromycin; CHL=chloramphenicol; CIP=ciprofloxacin; COL=colistin; CTA=cefotaxime; GEN=gentamicin; M

ESBL=extended-spectrum beta-lactamase-producing *E. coli*

GEN	MER	NAL	SME	CTZ	TET	TIG	TRI
1	<=0.03	64	16	>8	>32	<=0.25	<=0.25
<=0.5	<=0.03	>64	>512	2	>32	<=0.25	>16
1	<=0.03	>64	<=8	8	>32	0.5	<=0.25
<=0.5	<=0.03	>64	>512	4	<=2	0.5	>16
1	<=0.03	<=4	>512	8	32	0.5	>16
>16	<=0.03	>64	<=8	4	>32	<=0.25	<=0.25
1	<=0.03	<=4	>512	8	>32	0.5	>16
1	<=0.03	<=4	>512	2	4	<=0.25	>16
>16	<=0.03	>64	>512	8	>32	<=0.25	>16
1	<=0.03	<=4	<=8	4	<=2	<=0.25	<=0.25
1	<=0.03	<=4	>512	1	>32	<=0.25	>16
1	<=0.03	>64	>512	8	>32	0.5	>16
2	<=0.03	<=4	>512	4	<=2	0.5	>16
1	<=0.03	<=4	>512	1	<=2	0.5	>16
1	0.06	>64	>512	8	4	0.5	>16
<=0.5	0.06	>64	>512	8	8	0.5	>16
1	<=0.03	<=4	16	<=0.25	>32	<=0.25	0.5
<=0.5	<=0.03	<=4	<=8	<=0.25	<=2	0.5	<=0.25
>16	<=0.03	<=4	>512	<=0.25	>32	0.5	>16
2	<=0.03	<=4	<=8	<=0.25	<=2	0.5	<=0.25
>16	<=0.03	<=4	<=8	<=0.25	<=2	0.5	<=0.25
1	<=0.03	<=4	<=8	<=0.25	<=2	0.5	<=0.25
<=0.5	<=0.03	<=4	<=8	<=0.25	<=2	<=0.25	<=0.25
1	<=0.03	<=4	<=8	<=0.25	<=2	<=0.25	<=0.25
1	<=0.03	<=4	<=8	<=0.25	<=2	<=0.25	<=0.25
1	<=0.03	<=4	<=8	<=0.25	<=2	<=0.25	<=0.25
<=0.5	<=0.03	<=4	<=8	<=0.25	<=2	<=0.25	<=0.25
1	<=0.03	<=4	>512	<=0.25	<=2	<=0.25	>16
1	<=0.03	<=4	<=8	<=0.25	<=2	<=0.25	<=0.25
1	<=0.03	>64	<=8	8	<=2	<=0.25	<=0.25
1	<=0.03	<=4	>512	4	<=2	<=0.25	>16
1	<=0.03	<=4	>512	1	>32	0.5	>16
1	<=0.03	>64	<=8	2	>32	<=0.25	>16
4	<=0.03	<=4	>512	2	<=2	<=0.25	>16
1	<=0.03	>64	>512	8	>32	<=0.25	>16
1	<=0.03	<=4	<=8	<=0.25	<=2	<=0.25	<=0.25
<=0.5	<=0.03	<=4	<=8	<=0.25	<=2	<=0.25	<=0.25
1	<=0.03	64	>512	<=0.25	<=2	<=0.25	>16
2	<=0.03	>64	>512	<=0.25	<=2	<=0.25	>16
<=0.5	<=0.03	<=4	<=8	<=0.25	<=2	<=0.25	<=0.25
1	<=0.03	<=4	<=8	<=0.25	>32	<=0.25	<=0.25
1	<=0.03	<=4	<=8	<=0.25	<=2	<=0.25	<=0.25
1	<=0.03	<=4	<=8	<=0.25	4	<=0.25	<=0.25
1	<=0.03	<=4	>512	<=0.25	>32	<=0.25	>16
>16	<=0.03	64	>512	<=0.25	>32	<=0.25	>16
<=0.5	<=0.03	<=4	<=8	<=0.25	<=2	<=0.25	<=0.25
2	<=0.03	64	<=8	<=0.25	<=2	<=0.25	<=0.25
1	<=0.03	<=4	<=8	<=0.25	<=2	<=0.25	<=0.25

1	<=0.03	<=4	<=8	<=0.25	<=2	<=0.25	<=0.25
2	<=0.03	<=4	<=8	<=0.25	<=2	<=0.25	<=0.25
>16	0.06	>64	>512	8	4	<=0.25	>16
1	<=0.03	>64	>512	2	>32	<=0.25	>16
1	<=0.03	>64	>512	4	>32	<=0.25	>16
16	<=0.03	>64	>512	1	>32	<=0.25	>16
<=0.5	<=0.03	>64	>512	2	>32	<=0.25	>16
<=0.5	<=0.03	<=4	>512	4	<=2	<=0.25	>16
<=0.5	<=0.03	<=4	>512	2	4	<=0.25	>16
1	<=0.03	<=4	>512	2	4	<=0.25	>16
1	<=0.03	>64	<=8	<=0.25	<=2	<=0.25	<=0.25
1	-	>64	>512	>8	>32	<=0.25	>16
1	4	>64	>512	>8	>32	<=0.25	>16
1	<=0.03	>64	<=8	>8	4	<=0.25	<=0.25
1	<=0.03	>64	>512	8	>32	<=0.25	>16
1	<=0.03	>64	>512	4	>32	<=0.25	>16
<=0.5	<=0.03	>64	>512	2	<=2	0.5	>16
1	<=0.03	<=4	>512	1	>32	<=0.25	>16
1	<=0.03	>64	>512	2	>32	<=0.25	>16
1	<=0.03	<=4	>512	2	>32	<=0.25	>16
1	<=0.03	>64	>512	4	>32	<=0.25	>16
1	<=0.03	>64	>512	4	>32	<=0.25	>16
1	<=0.03	<=4	>512	4	<=2	<=0.25	>16
1	<=0.03	<=4	<=8	2	<=2	<=0.25	<=0.25
16	<=0.03	>64	>512	4	>32	<=0.25	>16
1	<=0.03	>64	>512	2	>32	<=0.25	>16
<=0.5	<=0.03	8	<=8	>8	<=2	<=0.25	<=0.25
1	<=0.03	<=4	>512	2	<=2	<=0.25	>16
>16	<=0.03	>64	>512	4	<=2	<=0.25	>16
1	<=0.03	<=4	<=8	<=0.25	<=2	<=0.25	<=0.25
1	<=0.03	<=4	<=8	<=0.25	<=2	<=0.25	<=0.25
<=0.5	<=0.03	<=4	<=8	<=0.25	<=2	<=0.25	<=0.25
<=0.5	<=0.03	8	>512	<=0.25	<=2	<=0.25	>16
1	<=0.03	>64	<=8	<=0.25	<=2	<=0.25	<=0.25
1	<=0.03	<=4	>512	<=0.25	>32	0.5	>16
1	<=0.03	<=4	<=8	<=0.25	4	<=0.25	<=0.25
2	<=0.03	<=4	<=8	<=0.25	<=2	<=0.25	<=0.25
1	<=0.03	<=4	<=8	<=0.25	<=2	<=0.25	<=0.25
2	<=0.03	<=4	<=8	<=0.25	<=2	<=0.25	<=0.25
<=0.5	<=0.03	<=4	>512	<=0.25	32	<=0.25	<=0.25
1	<=0.03	<=4	>512	<=0.25	>32	<=0.25	>16
1	<=0.03	<=4	<=8	<=0.25	>32	<=0.25	<=0.25
1	<=0.03	<=4	<=8	<=0.25	>32	<=0.25	<=0.25
1	<=0.03	<=4	<=8	<=0.25	>32	<=0.25	<=0.25
<=0.5	<=0.03	<=4	<=8	<=0.25	4	<=0.25	<=0.25
<=0.5	<=0.03	>64	>512	>8	32	<=0.25	>16
1	<=0.03	>64	>512	8	>32	<=0.25	>16
<=0.5	<=0.03	>64	>512	8	>32	<=0.25	>16
>16	8	>64	>512	>8	>32	<=0.25	>16
<=0.5	0.06	<=4	>512	2	>32	<=0.25	>16
>16	<=0.03	<=4	>512	-	>32	<=0.25	>16

>16	<=0.03	<=4	>512	-	>32	<=0.25	>16
1	<=0.03	<=4	<=8	<=0.25	<=2	<=0.25	0.5
<=0.5	<=0.03	<=4	<=8	0.5	4	<=0.25	<=0.25
1	<=0.03	>64	>512	8	>32	0.5	>16
<=0.5	<=0.03	>64	>512	>8	>32	0.5	>16
1	<=0.03	16	16	8	8	<=0.25	<=0.25
1	<=0.03	<=4	16	8	<=2	<=0.25	0.5
1	<=0.03	<=4	>512	4	<=2	<=0.25	>16
1	<=0.03	8	<=8	2	<=2	<=0.25	<=0.25
<=0.5	<=0.03	<=4	16	2	4	<=0.25	<=0.25
1	<=0.03	16	<=8	8	4	<=0.25	<=0.25
1	<=0.03	<=4	<=8	2	<=2	<=0.25	<=0.25
1	<=0.03	<=4	>512	4	>32	0.5	<=0.25
1	<=0.03	<=4	<=8	2	<=2	<=0.25	<=0.25
<=0.5	<=0.03	<=4	>512	2	>32	<=0.25	>16
2	<=0.03	8	>512	2	>32	<=0.25	>16
1	<=0.03	<=4	<=8	1	>32	<=0.25	0.5
1	<=0.03	>64	<=8	2	<=2	<=0.25	>16
2	<=0.03	>64	>512	8	>32	0.5	>16
1	<=0.03	>64	>512	8	>32	0.5	>16
2	<=0.03	>64	>512	4	>32	<=0.25	>16
1	<=0.03	>64	>512	4	>32	<=0.25	>16
1	<=0.03	>64	>512	2	>32	<=0.25	>16
1	<=0.03	>64	>512	2	<=2	<=0.25	>16
4	<=0.03	<=4	>512	2	4	0.5	>16
1	<=0.03	<=4	16	2	>32	0.5	>16
1	<=0.03	<=4	>512	8	>32	0.5	>16
1	<=0.03	<=4	>512	8	>32	<=0.25	>16
1	<=0.03	<=4	>512	1	>32	0.5	>16
<=0.5	<=0.03	<=4	>512	2	>32	0.5	>16
<=0.5	<=0.03	<=4	>512	4	>32	<=0.25	>16
<=0.5	<=0.03	<=4	>512	2	<=2	0.5	<=0.25
1	<=0.03	<=4	<=8	2	>32	<=0.25	<=0.25
<=0.5	<=0.03	<=4	<=8	2	>32	<=0.25	<=0.25
<=0.5	<=0.03	<=4	>512	2	<=2	<=0.25	<=0.25
<=0.5	<=0.03	<=4	<=8	2	>32	<=0.25	<=0.25
1	<=0.03	<=4	<=8	8	<=2	<=0.25	<=0.25
1	<=0.03	<=4	>512	2	>32	0.5	>16
1	<=0.03	<=4	>512	2	<=2	<=0.25	<=0.25
1	<=0.03	<=4	<=8	2	<=2	0.5	<=0.25
1	<=0.03	<=4	>512	2	>32	0.5	>16
2	<=0.03	<=4	>512	4	>32	1	>16
1	<=0.03	<=4	>512	4	>32	0.5	>16
1	<=0.03	<=4	>512	2	>32	0.5	>16
<=0.5	<=0.03	<=4	>512	1	>32	0.5	>16
1	<=0.03	<=4	<=8	1	<=2	0.5	<=0.25
<=0.5	<=0.03	<=4	>512	<=0.25	<=2	<=0.25	<=0.25
<=0.5	<=0.03	128	16	<=0.5	<=2	<=0.25	0.5
<=0.5	<=0.03	16	>1024	<=0.5	<=2	<=0.25	1
>32	<=0.03	<=4	>1024	<=0.5	>64	<=0.25	>32
1	<=0.03	8	<=8	<=0.5	>64	<=0.25	<=0.25

1	<=0.03	<=4	16	<=0.5	64	<=0.25	<=0.25
1	<=0.03	<=4	16	<=0.5	<=2	<=0.25	0.5
<=0.5	<=0.03	<=4	16	<=0.5	<=2	<=0.25	0.5
<=0.5	<=0.03	<=4	<=8	<=0.5	<=2	<=0.25	0.5
1	<=0.03	<=4	16	<=0.5	<=2	<=0.25	<=0.25
<=0.5	<=0.03	<=4	<=8	<=0.5	4	<=0.25	1
<=0.5	<=0.03	<=4	>1024	<=0.5	64	<=0.25	>32
1	<=0.03	<=4	<=8	<=0.5	>64	<=0.25	<=0.25
<=0.5	<=0.03	<=4	16	<=0.5	<=2	<=0.25	<=0.25
1	<=0.03	<=4	16	<=0.5	<=2	<=0.25	<=0.25
<=0.5	<=0.03	<=4	<=8	<=0.5	>64	<=0.25	<=0.25
>32	<=0.03	16	>1024	<=0.5	>64	<=0.25	>32
<=0.5	<=0.03	<=4	128	<=0.5	64	<=0.25	>32
1	<=0.03	<=4	16	<=0.5	64	<=0.25	0.5
<=0.5	<=0.03	<=4	>1024	<=0.5	64	<=0.25	0.5
1	<=0.03	<=4	32	<=0.5	<=2	<=0.25	<=0.25
<=0.5	<=0.03	<=4	16	<=0.5	<=2	<=0.25	0.5
1	0.06	<=4	16	<=0.5	<=2	<=0.25	0.5
1	<=0.03	<=4	32	<=0.5	<=2	<=0.25	1
<=0.5	<=0.03	<=4	<=8	<=0.5	<=2	<=0.25	<=0.25
<=0.5	<=0.03	<=4	<=8	<=0.5	<=2	<=0.25	<=0.25
<=0.5	<=0.03	<=4	<=8	<=0.5	<=2	<=0.25	<=0.25
<=0.5	<=0.03	<=4	<=8	<=0.5	64	<=0.25	<=0.25
2	<=0.03	<=4	<=8	<=0.5	4	<=0.25	<=0.25
<=0.5	<=0.03	8	>1024	<=0.5	>64	<=0.25	>32
<=0.5	<=0.03	8	<=8	<=0.5	<=2	<=0.25	<=0.25
<=0.5	<=0.03	<=4	<=8	<=0.5	>64	<=0.25	<=0.25
<=0.5	<=0.03	<=4	>1024	<=0.5	<=2	<=0.25	>32
1	<=0.03	<=4	<=8	<=0.5	<=2	<=0.25	<=0.25
<=0.5	<=0.03	<=4	>1024	<=0.5	64	0.5	>32
1	<=0.03	<=4	>1024	<=0.5	>64	0.5	<=0.25
1	<=0.03	8	<=8	<=0.5	8	<=0.25	0.5
<=0.5	<=0.03	>128	>1024	<=0.5	>64	0.5	>32
<=0.5	<=0.03	<=4	>1024	<=0.5	<=2	<=0.25	>32
<=0.5	<=0.03	<=4	<=8	<=0.5	64	<=0.25	<=0.25
2	<=0.03	>128	>1024	<=0.5	32	<=0.25	>32
<=0.5	<=0.03	<=4	<=8	<=0.5	<=2	<=0.25	<=0.25
1	<=0.03	<=4	<=8	<=0.5	<=2	<=0.25	<=0.25
<=0.5	<=0.03	<=4	<=8	<=0.5	>64	<=0.25	<=0.25
<=0.5	<=0.03	<=4	>1024	<=0.5	>64	<=0.25	>32
<=0.5	<=0.03	<=4	<=8	<=0.5	<=2	<=0.25	<=0.25
<=0.5	<=0.03	<=4	<=8	<=0.5	<=2	<=0.25	<=0.25
<=0.5	<=0.03	<=4	<=8	<=0.5	<=2	<=0.25	<=0.25
1	<=0.03	<=4	>512	<=0.25	<=2	<=0.25	<=0.25
<=0.5	<=0.03	<=4	>512	2	>32	<=0.25	>16
<=0.5	<=0.03	<=4	<=8	>8	<=2	<=0.25	<=0.25
>16	<=0.03	<=4	>512	2	>32	<=0.25	<=0.25
1	<=0.03	<=4	<=8	<=0.25	>32	<=0.25	0.5
<=0.5	<=0.03	<=4	16	<=0.5	>64	<=0.25	1
<=0.5	<=0.03	<=4	>1024	<=0.5	64	<=0.25	>32
<=0.5	<=0.03	<=4	<=8	<=0.5	<=2	<=0.25	<=0.25

>32	<=0.03	<=4	>1024	<=0.5	32	<=0.25	>32
2	<=0.03	64	>1024	1	>64	<=0.25	>32
<=0.5	0.06	<=4	>1024	4	4	<=0.25	>32
1	<=0.03	<=4	>1024	<=0.5	<=2	<=0.25	>32
32	<=0.03	<=4	>1024	<=0.5	>64	<=0.25	>32
1	<=0.03	<=4	16	2	<=2	<=0.25	<=0.25
<=0.5	<=0.03	128	16	8	>64	<=0.25	<=0.25
<=0.5	<=0.03	16	>1024	<=0.5	32	<=0.25	>32
<=0.5	<=0.03	128	>1024	8	>64	<=0.25	8
<=0.5	<=0.03	<=4	>1024	2	<=2	<=0.25	>32
1	<=0.03	<=4	>1024	<=0.5	>64	<=0.25	>32
<=0.5	<=0.03	<=4	>1024	<=0.5	64	<=0.25	>32
<=0.5	<=0.03	<=4	16	<=0.5	<=2	<=0.25	0.5
<=0.5	<=0.03	<=4	16	<=0.5	<=2	<=0.25	0.5
1	<=0.03	<=4	>1024	<=0.5	<=2	<=0.25	>32
<=0.5	<=0.03	<=4	16	<=0.5	<=2	<=0.25	>32
<=0.5	<=0.03	<=4	>1024	4	<=2	<=0.25	1
1	<=0.03	<=4	>1024	2	<=2	<=0.25	>32
<=0.5	<=0.03	<=4	64	2	<=2	<=0.25	<=0.25
2	<=0.03	<=4	<=8	<=0.5	<=2	<=0.25	<=0.25
<=0.5	<=0.03	<=4	32	<=0.5	<=2	<=0.25	0.5
<=0.5	<=0.03	>128	>1024	2	>64	<=0.25	>32
2	<=0.03	<=4	16	2	<=2	<=0.25	<=0.25
<=0.5	<=0.03	<=4	16	<=0.5	<=2	<=0.25	<=0.25
<=0.5	<=0.03	<=4	>1024	2	<=2	<=0.25	>32
>32	<=0.03	<=4	>1024	<=0.5	64	<=0.25	>32
<=0.5	<=0.03	<=4	16	2	<=2	<=0.25	<=0.25
1	<=0.03	8	32	8	>64	<=0.25	<=0.25
32	<=0.03	>128	>1024	>8	64	<=0.25	>32
1	<=0.03	<=4	16	<=0.5	4	<=0.25	1
<=0.5	<=0.03	<=4	16	<=0.5	<=2	<=0.25	<=0.25
1	0.06	<=4	>1024	<=0.5	>64	<=0.25	>32
1	<=0.03	<=4	16	<=0.5	<=2	<=0.25	<=0.25
1	<=0.03	<=4	16	2	>64	<=0.25	<=0.25
<=0.5	<=0.03	>128	>1024	8	>64	<=0.25	>32
2	<=0.03	<=4	16	<=0.5	4	<=0.25	1
<=0.5	<=0.03	<=4	>1024	1	<=2	<=0.25	>32
<=0.5	<=0.03	<=4	16	<=0.5	<=2	<=0.25	0.5
1	<=0.03	<=4	16	<=0.5	<=2	<=0.25	1
1	<=0.03	<=4	16	<=0.5	<=2	<=0.25	<=0.25
<=0.5	<=0.03	>128	>1024	<=0.5	<=2	<=0.25	>32
<=0.5	<=0.03	<=4	16	<=0.5	4	<=0.25	0.5
<=0.5	<=0.03	<=4	>1024	2	<=2	<=0.25	>32
<=0.5	<=0.03	<=4	<=8	<=0.5	>64	<=0.25	<=0.25
<=0.5	<=0.03	<=4	>1024	<=0.5	64	0.5	>32
<=0.5	<=0.03	<=4	>1024	<=0.5	>64	<=0.25	>32
<=0.5	<=0.03	<=4	<=8	<=0.5	64	<=0.25	<=0.25
<=0.5	<=0.03	<=4	>1024	<=0.5	<=2	<=0.25	>32
<=0.5	<=0.03	<=4	>1024	1	<=2	<=0.25	>32
<=0.5	<=0.03	<=4	<=8	<=0.5	4	0.5	<=0.25
<=0.5	<=0.03	<=4	<=8	<=0.5	<=2	<=0.25	<=0.25

<=0.5	<=0.03	>128	<=8	8	<=2	<=0.25	<=0.25
2	<=0.03	<=4	16	2	>64	<=0.25	<=0.25
<=0.5	<=0.03	<=4	<=8	<=0.5	4	<=0.25	<=0.25
<=0.5	<=0.03	<=4	<=8	<=0.5	<=2	<=0.25	<=0.25
<=0.5	<=0.03	<=4	<=8	<=0.5	<=2	<=0.25	<=0.25
<=0.5	<=0.03	<=4	<=8	<=0.5	<=2	<=0.25	<=0.25
<=0.5	<=0.03	<=4	>1024	<=0.5	64	<=0.25	>32
<=0.5	<=0.03	<=4	<=8	<=0.5	<=2	<=0.25	0.5
<=0.5	<=0.03	<=4	<=8	<=0.5	<=2	<=0.25	<=0.25
<=0.5	<=0.03	<=4	>1024	<=0.5	64	0.5	<=0.25
<=0.5	<=0.03	<=4	<=8	<=0.5	64	<=0.25	<=0.25
<=0.5	<=0.03	<=4	<=8	<=0.5	<=2	<=0.25	0.5
<=0.5	<=0.03	<=4	>1024	2	64	<=0.25	>32
2	<=0.03	<=4	<=8	<=0.5	64	<=0.25	<=0.25
<=0.5	<=0.03	<=4	>1024	<=0.5	>64	<=0.25	>32
<=0.5	<=0.03	<=4	<=8	<=0.5	<=2	<=0.25	<=0.25
<=0.5	<=0.03	<=4	<=8	<=0.5	<=2	<=0.25	<=0.25
<=0.5	<=0.03	128	>1024	<=0.5	>64	<=0.25	>32
1	<=0.03	>128	>1024	4	>64	<=0.25	>32
<=0.5	<=0.03	8	<=8	<=0.5	4	0.5	<=0.25
<=0.5	<=0.03	8	<=8	<=0.5	4	<=0.25	<=0.25
2	<=0.03	<=4	16	<=0.5	<=2	<=0.25	0.5
<=0.5	<=0.03	<=4	>1024	4	<=2	<=0.25	>32
<=0.5	<=0.03	<=4	>1024	2	<=2	<=0.25	>32
<=0.5	<=0.03	<=4	<=8	<=0.5	<=2	<=0.25	0.5
1	<=0.03	<=4	>1024	4	<=2	0.5	<=0.25
<=0.5	<=0.03	<=4	>1024	1	64	0.5	>32
<=0.5	<=0.03	>128	<=8	>8	>64	<=0.25	0.5
1	<=0.03	<=4	>1024	<=0.5	>64	<=0.25	>32
<=0.5	<=0.03	<=4	<=8	<=0.5	>64	<=0.25	<=0.25
<=0.5	<=0.03	<=4	>1024	1	<=2	<=0.25	>32
32	<=0.03	<=4	32	2	<=2	<=0.25	<=0.25
<=0.5	<=0.03	>128	>1024	2	8	<=0.25	>32
<=0.5	<=0.03	<=4	>1024	1	64	0.5	>32
<=0.5	<=0.03	>128	>1024	1	>64	0.5	<=0.25
2	<=0.03	<=4	>1024	2	<=2	<=0.25	>32
1	<=0.03	<=4	>1024	2	<=2	<=0.25	>32
1	<=0.03	<=4	>1024	1	<=2	<=0.25	<=0.25
32	<=0.03	>128	>1024	>8	>64	<=0.25	>32
32	<=0.03	<=4	<=8	2	>64	<=0.25	<=0.25
<=0.5	<=0.03	<=4	>1024	2	64	<=0.25	>32
<=0.5	<=0.03	<=4	<=8	<=0.5	>64	0.5	<=0.25
1	<=0.03	<=4	>1024	<=0.5	<=2	0.5	>32
<=0.5	<=0.03	<=4	16	2	>64	<=0.25	<=0.25
<=0.5	<=0.03	<=4	<=8	8	>64	<=0.25	<=0.25
1	<=0.03	<=4	32	2	<=2	<=0.25	<=0.25
1	<=0.03	>64	>512	>8	4	<=0.25	>16
<=0.5	<=0.03	<=4	>512	2	>32	<=0.25	>16
<=0.5	<=0.03	<=4	>512	2	>32	<=0.25	>16
<=0.5	<=0.03	<=4	>512	4	>32	0.5	>16
2	<=0.03	<=4	>512	2	>32	<=0.25	<=0.25

1	<=0.03	>64	<=8	2	<=2	<=0.25	<=0.25
1	<=0.03	<=4	>512	2	<=2	<=0.25	>16
<=0.5	<=0.03	>64	>512	4	>32	<=0.25	>16
2	<=0.03	<=4	>512	2	<=2	<=0.25	>16
2	<=0.03	>64	>512	>8	<=2	<=0.25	>16
1	<=0.03	64	>512	1	<=2	<=0.25	>16
<=0.5	<=0.03	<=4	>512	1	<=2	<=0.25	>16
<=0.5	<=0.03	>64	>512	2	>32	<=0.25	<=0.25
>16	<=0.03	>64	>512	>8	>32	<=0.25	>16
1	<=0.03	>64	<=8	>8	4	<=0.25	<=0.25
2	<=0.03	>64	>512	>8	>32	<=0.25	>16
1	<=0.03	>64	>512	8	>32	<=0.25	>16
>16	1	64	>512	2	4	<=0.25	>16
4	0.5	>64	<=8	4	<=2	<=0.25	>16
1	<=0.03	>64	>512	8	>32	<=0.25	>16
4	<=0.03	>64	>512	4	<=2	<=0.25	>16
2	<=0.03	32	<=8	8	4	0.5	<=0.25
<=0.5	<=0.03	>64	<=8	2	4	<=0.25	<=0.25
-	0.06	>64	<=8	2	<=2	<=0.25	<=0.25
<=0.5	>16	>64	>512	>8	>32	0.5	>16
1	<=0.03	>64	>512	>8	<=2	<=0.25	>16
1	<=0.03	>64	<=8	0.5	>32	<=0.25	>16
>16	<=0.03	>64	>512	8	<=2	<=0.25	>16
1	<=0.03	>64	<=8	4	>32	<=0.25	>16
1	<=0.03	>64	>512	>8	>32	<=0.25	>16
<=0.5	<=0.03	>64	<=8	4	<=2	<=0.25	<=0.25
1	<=0.03	>64	>512	4	<=2	<=0.25	<=0.25
1	<=0.03	>64	<=8	4	>32	<=0.25	<=0.25
>16	<=0.03	>64	>512	0.5	>32	<=0.25	>16
2	<=0.03	>64	>512	2	>32	0.5	>16
1	<=0.03	>64	<=8	2	<=2	<=0.25	<=0.25
16	<=0.03	>64	>512	2	4	<=0.25	>16
1	<=0.03	>64	<=8	>8	>32	<=0.25	<=0.25
2	<=0.03	>64	<=8	4	<=2	<=0.25	<=0.25
<=0.5	<=0.03	>64	>512	>8	>32	<=0.25	>16
2	<=0.03	>64	<=8	>8	>32	<=0.25	>16
<=0.5	<=0.03	>64	>512	>8	4	<=0.25	>16
<=0.5	<=0.03	>64	>512	>8	>32	<=0.25	>16
>16	<=0.03	>64	>512	>8	>32	<=0.25	>16
1	<=0.03	>64	>512	>8	>32	<=0.25	>16
1	<=0.03	>64	>512	>8	>32	<=0.25	>16
1	<=0.03	>64	>512	2	>32	0.5	>16
1	<=0.03	>64	>512	4	>32	<=0.25	>16
2	<=0.03	>64	>512	8	>32	<=0.25	>16
1	<=0.03	>64	16	4	4	<=0.25	<=0.25
1	<=0.03	8	<=8	1	8	<=0.25	0.5
<=0.5	<=0.03	>64	>512	2	>32	<=0.25	>16
1	0.06	>64	64	>8	>32	<=0.25	0.5
1	<=0.03	>64	>512	4	>32	<=0.25	>16
1	<=0.03	>64	>512	8	4	<=0.25	>16
<=0.5	<=0.03	>64	>512	4	>32	<=0.25	>16

<=0.5	<=0.03	>64	>512	2	<=2	<=0.25	>16
>16	<=0.03	>64	32	>8	>32	<=0.25	0.5
1	<=0.03	32	>512	>8	4	<=0.25	>16
1	0.06	>64	>512	2	>32	<=0.25	>16
1	<=0.03	<=4	>512	4	>32	<=0.25	>16
1	<=0.03	>64	>512	2	<=2	<=0.25	>16
1	<=0.03	>64	<=8	8	<=2	<=0.25	0.5
1	<=0.03	>64	>512	8	>32	<=0.25	>16
2	<=0.03	>64	>512	2	>32	<=0.25	>16
2	<=0.03	>64	>512	>8	>32	<=0.25	>16
1	<=0.03	>64	>512	2	>32	<=0.25	>16
<=0.5	<=0.03	<=4	<=8	>8	<=2	<=0.25	<=0.25
2	<=0.03	>64	16	<=0.25	<=2	<=0.25	0.5
<=0.5	<=0.03	>64	>512	>8	>32	<=0.25	>16
2	<=0.03	<=4	>512	2	<=2	<=0.25	>16
1	<=0.03	<=4	>512	<=0.25	>32	0.5	>16
1	<=0.03	>64	16	<=0.25	4	<=0.25	<=0.25
1	<=0.03	<=4	>512	<=0.25	>32	<=0.25	>16
<=0.5	<=0.03	>64	>512	<=0.25	<=2	<=0.25	>16
<=0.5	<=0.03	<=4	<=8	<=0.25	<=2	<=0.25	<=0.25
<=0.5	<=0.03	>64	>512	<=0.25	<=2	<=0.25	>16
1	<=0.03	>64	<=8	0.5	<=2	<=0.25	<=0.25
4	0.06	16	>512	0.5	>32	<=0.25	>16
1	<=0.03	>64	>512	<=0.25	>32	<=0.25	>16
1	<=0.03	>64	>512	<=0.25	>32	<=0.25	>16
1	<=0.03	>64	<=8	0.5	>32	<=0.25	<=0.25
1	<=0.03	>64	>512	<=0.25	>32	<=0.25	>16
1	<=0.03	<=4	<=8	<=0.25	<=2	<=0.25	<=0.25
1	<=0.03	>64	>512	<=0.25	>32	<=0.25	>16
1	<=0.03	<=4	>512	<=0.25	>32	<=0.25	>16
<=0.5	<=0.03	<=4	<=8	<=0.25	<=2	<=0.25	<=0.25
2	<=0.03	<=4	>512	<=0.25	<=2	<=0.25	>16
<=0.5	<=0.03	<=4	<=8	<=0.25	<=2	<=0.25	<=0.25
1	<=0.03	<=4	<=8	-	<=2	<=0.25	<=0.25
1	<=0.03	>64	<=8	<=0.25	<=2	<=0.25	>16
>16	<=0.03	>64	>512	1	>32	<=0.25	0.5
1	<=0.03	<=4	>512	<=0.25	4	<=0.25	<=0.25
2	<=0.03	<=4	32	<=0.25	<=2	<=0.25	<=0.25
1	<=0.03	>64	<=8	0.5	<=2	<=0.25	<=0.25

MER=meropenem; NAL=nalidixic acid; SME=sulfamethoxazole; CTZ=ceftazidime; TET=tetracycline; TIG=tigecycline; TRI=trimethoprim

ı.
