# Supplementary material for: Biocide Susceptibility and Antimicrobial Resistance of Escherichia coli Isolated from Swine Feces, Pork Meat and Humans in Germany
Source: Antibiotics (Basel). 2023 Apr 27;12(5):823. doi: 10.3390/antibiotics12050823 (PMC10215396; doi:10.3390/antibiotics12050823)
Supplement: Supplementary file 1 [file antibiotics-12-00823-s001.zip › antibiotics-2359558-supplementary/Table S5.pdf]

**Table S5a.** Significant positive correlation coefficients ($r_s$) between biocides and antimicrobials in *E. coli* of hur

| Origin of isolates | $r_s$ | GDA $_{MIC}$ | GDA $_{MBC}$ | CHG $_{MIC}$ | CHG $_{MBC}$ | BAC $_{MIC}$ | BAC $_{MBC}$ |
|---|---|---|---|---|---|---|---|
| Swine feces | AZI | - | - | 0.289** | - | - | - |
| | GEN | 0.246* | - | - | - | - | - |
| | TET | - | - | 0.291** | 0.276** | - | - |
| Pork meat | TET | - | - | - | 0.344** | - | - |
| | COL | - | - | 0.212* | 0.201* | - | - |
| | NAL | - | - | 0.225* | 0.245* | - | - |
| | CTZ | - | - | 0.370** | 0.228* | - | - |
| Voluntary donors | AMP | - | - | - | - | 0.216* | 0.301** |
| | AZI | - | - | - | - | 0.311** | 0.247* |
| | CHL | - | - | 0.284** | - | 0.297** | 0.317** |
| | CIP | - | - | - | - | 0.274** | 0.238* |
| | COL | - | - | - | - | - | - |
| | CTA | - | - | - | - | 0.230* | 0.327** |
| | MER | - | - | - | - | - | 0.202* |
| | GEN | - | - | 0.212* | - | - | - |
| | NAL | - | - | - | - | 0.215* | 0.213* |
| | SME | - | - | - | - | 0.216* | 0.213* |
| | CTZ | - | - | - | - | 0.312** | 0.376** |
| | TET | - | - | 0.219* | 0.310** | - | - |
| | TIG | - | - | - | - | 0.264** | 0.259* |
| | TRI | - | - | - | - | - | 0.228* |
| Inpatients | AMP | - | - | - | - | - | - |
| | CHL | - | - | - | - | - | - |
| | CIP | - | - | - | 0.224* | 0.202* | - |
| | NAL | - | - | - | 0.302** | 0.208* | - |
| | TET | - | - | - | - | 0.205* | - |
| | COL | - | - | - | - | - | - |
| | AZI | - | - | - | 0.213* | - | - |
| | CTA | - | - | - | - | - | - |

**Table S5b.** Significant positive correlation coefficients ($r_s$) between biocides and antimicrobials in non-ESBL an

| Origin of isolates | $r_s$ | GDA $_{MIC}$ | GDA $_{MBC}$ | CHG $_{MIC}$ | CHG $_{MBC}$ | BAC $_{MIC}$ | BAC $_{MBC}$ |
|---|---|---|---|---|---|---|---|
| ESBL | AZI | - | - | 0.165* | 0.165* | 0.140* | 0.162* |
| | CIP | - | - | 0.201** | 0.259** | 0.187** | - |
| | MER | 0.160* | 0.141* | - | - | - | - |
| | NAL | - | - | 0.193** | 0.254** | 0.201** | - |
| | TET | - | - | 0.180** | 0.196** | - | - |
| | TIG | - | - | - | - | - | 0.174* |
| | GEN | - | - | 0.161* | - | 0.161* | - |
| | CTZ | - | - | - | - | 0.194** | - |
| | CHL | - | - | - | - | - | - |
| non-ESBL | AMP | - | - | - | - | 0.227** | 0.181* |
| | AZI | - | - | - | - | 0.231** | - |
| | CIP | 0.154* | - | 0.168* | 0.227** | - | - |
| | GEN | 0.185* | - | 0.286** | 0.222** | - | - |
| | NAL | - | - | 0.161* | 0.251** | - | - |
| | SME | - | - | - | - | 0.181* | 0.173* |
| | CTZ | - | - | - | - | 0.228** | 0.307** |
| | TET | - | - | 0.233** | 0.249** | - | - |
| | TMP | - | - | - | - | 0.208** | 0.219** |
| | CTA | - | - | - | - | - | - |

GDA=glutaraldehyde; CHG=chlorhexidine digluconate; BAC=benzalkonium chloride; OCT=octenidine dihydrochloride; IPA=isopropanol;

AMP=ampicillin; CTA=cefotaxime; CTZ=ceftazidime; MER=meropenem; NAL=nalidixic acid; CIP=ciprofloxacin; GEN=gentamicin; TET= tet

MIC=minimum inhibitory concentration; MBC=minimum bactericidal concentration

* Correlation is significant at the 0.05 level; ** Correlation is significant at the 0.01 level.

nan and porcine origin.

| OCT $_{MIC}$ | OCT $_{MBC}$ | IPA $_{MIC}$ | IPA $_{MBC}$ | PCMC $_{MIC}$ | PCMC $_{MBC}$ | NaOCl $_{MIC}$ | NaOCl $_{MBC}$ |
|---|---|---|---|---|---|---|---|
| - | - | - | - | - | - | - | - |
| - | - | - | - | - | - | - | - |
| - | - | - | - | - | - | - | - |
| - | - | - | - | - | - | - | - |
| - | - | - | - | - | - | - | - |
| - | - | - | - | - | - | - | - |
| - | - | - | - | - | - | - | - |
| - | - | - | - | - | - | - | - |
| - | - | - | - | - | - | - | - |
| - | - | - | - | - | - | - | - |
| - | - | - | - | - | - | - | - |
| 0.203* | - | - | - | - | - | - | - |
| - | - | - | - | - | - | - | - |
| - | - | - | - | - | - | - | - |
| - | - | - | - | - | - | - | - |
| - | - | - | - | - | - | - | - |
| - | - | - | - | - | - | - | - |
| - | - | - | - | - | - | - | - |
| - | - | - | - | - | - | - | - |
| - | - | - | - | - | - | - | - |
| - | - | - | - | - | - | - | - |
| - | - | - | - | - | 0.278** | 0.237* | - |
| - | - | - | - | 0.273** | - | - | - |
| - | - | - | - | - | - | - | - |
| - | - | - | - | - | - | - | - |
| - | - | - | - | - | - | - | - |
| - | - | - | - | - | - | - | 0.202* |
| - | - | - | - | - | - | - | - |
| - | - | - | - | - | 0.282** | - | - |

d ESBL *E. coli*

| OCT $_{MIC}$ | OCT $_{MBC}$ | IPA $_{MIC}$ | IPA $_{MBC}$ | PCMC $_{MIC}$ | PCMC $_{MBC}$ | NaOCl $_{MIC}$ | NaOCl $_{MBC}$ |
|---|---|---|---|---|---|---|---|
| - | - | - | - | - | - | - | - |
| - | - | - | - | - | - | - | - |
| - | - | - | - | - | - | - | - |
| - | - | - | - | - | - | - | - |
| - | - | - | - | - | - | - | - |
| - | - | - | - | - | - | - | - |
| - | - | 0.157* | - | - | - | - | - |
| - | - | - | - | - | - | - | - |
| - | - | - | - | 0.165* | - | - | - |
| - | - | - | - | - | - | - | - |
| - | - | - | - | - | - | - | - |
| - | - | - | - | - | - | - | - |
| - | - | - | - | - | - | - | - |
| - | - | - | - | - | - | - | - |
| - | - | - | - | - | - | - | - |
| - | - | - | - | - | - | - | - |
| - | - | - | - | - | - | - | - |
| - | - | - | - | - | - | - | - |
| - | - | - | 0.155* | - | - | - | - |

NaOCl=sodium hypochlorite; PCMC=chlorocresol.

tracycline; TIG=tigecycline; COL=colistin; SME=sulfamethoxazole; TRI=trimethoprim; CHL=chloramphenicol; AZI=azithromycin.
